# Functional Tradeoffs Underpin Salinity-Driven Divergence in Microbial Community Composition

**DOI:** 10.1371/journal.pone.0089549

**Published:** 2014-02-27

**Authors:** Chris L. Dupont, John Larsson, Shibu Yooseph, Karolina Ininbergs, Johannes Goll, Johannes Asplund-Samuelsson, John P. McCrow, Narin Celepli, Lisa Zeigler Allen, Martin Ekman, Andrew J. Lucas, Åke Hagström, Mathangi Thiagarajan, Björn Brindefalk, Alexander R. Richter, Anders F. Andersson, Aaron Tenney, Daniel Lundin, Andrey Tovchigrechko, Johan A. A. Nylander, Daniel Brami, Jonathan H. Badger, Andrew E. Allen, Douglas B. Rusch, Jeff Hoffman, Erling Norrby, Robert Friedman, Jarone Pinhassi, J. Craig Venter, Birgitta Bergman

**Affiliations:** 1 Microbial and Environmental Genomics, J. Craig Venter Institute, San Diego, California, United States of America; 2 Department of Ecology, Environment and Plant Sciences, Stockholm University, Stockholm, Sweden; 3 Informatics Group, J. Craig Venter Institute, San Diego, California, United States of America; 4 Informatics Group, J. Craig Venter Institute, Rockville, Maryland, United States of America; 5 Marine Physical Laboratory, Scripps Institution of Oceanography, University of California San Diego, San Diego, California, United States of America; 6 Swedish Institute for the Marine Environment (SIME), University of Gothenburg, Gothenburg, Sweden; 7 KTH Royal Institute of Technology, Science for Life Laboratory, School of Biotechnology, Solna, Sweden; 8 Department of Biodiversity Informatics, Swedish Museum of Natural History, Stockholm, Sweden; 9 Center for History of Science, The Royal Swedish Academy of Sciences, Stockholm, Sweden; 10 Centre for Ecology and Evolution in Microbial Model Systems, Linnaeus University, Kalmar, Sweden; Catalan Institute for Water Research (ICRA), Spain

## Abstract

Bacterial community composition and functional potential change subtly across gradients in the surface ocean. In contrast, while there are significant phylogenetic divergences between communities from freshwater and marine habitats, the underlying mechanisms to this phylogenetic structuring yet remain unknown. We hypothesized that the functional potential of natural bacterial communities is linked to this striking divide between microbiomes. To test this hypothesis, metagenomic sequencing of microbial communities along a 1,800 km transect in the Baltic Sea area, encompassing a continuous natural salinity gradient from limnic to fully marine conditions, was explored. Multivariate statistical analyses showed that salinity is the main determinant of dramatic changes in microbial community composition, but also of large scale changes in core metabolic functions of bacteria. Strikingly, genetically and metabolically different pathways for key metabolic processes, such as respiration, biosynthesis of quinones and isoprenoids, glycolysis and osmolyte transport, were differentially abundant at high and low salinities. These shifts in functional capacities were observed at multiple taxonomic levels and within dominant bacterial phyla, while bacteria, such as SAR11, were able to adapt to the entire salinity gradient. We propose that the large differences in central metabolism required at high and low salinities dictate the striking divide between freshwater and marine microbiomes, and that the ability to inhabit different salinity regimes evolved early during bacterial phylogenetic differentiation. These findings significantly advance our understanding of microbial distributions and stress the need to incorporate salinity in future climate change models that predict increased levels of precipitation and a reduction in salinity.

## Introduction

Metagenomic surveys of the surface ocean reveal a continuum in bacterial community composition with phylogenetic variations at the genus or family level [1]–[4] while phylogenetic marker surveys of salinity gradients have highlighted changes at higher taxonomic levels [5]–[10]. Despite large population sizes and high dispersal rates, the boundary between freshwater and marine environments appears to constitute an almost insurmountable transition barrier for bacteria [8], yet the underlying reasons have remained enigmatic. A few studies have compared genomic characteristics of microbial communities at a limited number of salinities, with subtle changes observed in functional gene repertoires [11]–[13]. While highly significant, these studies examined disconnected microbial communities and used different extraction procedures, which limit comparisons [14].. To surpass such shortcomings, we examined functional adaptations to salinity along a natural continuous gradient, spanning the freshwater to marine range, which allowed direct comparative metagenomic analyses, and used robust multivariate statistical methods, fully incorporating potentially co-varying environmental variables.

**Figure 1:**
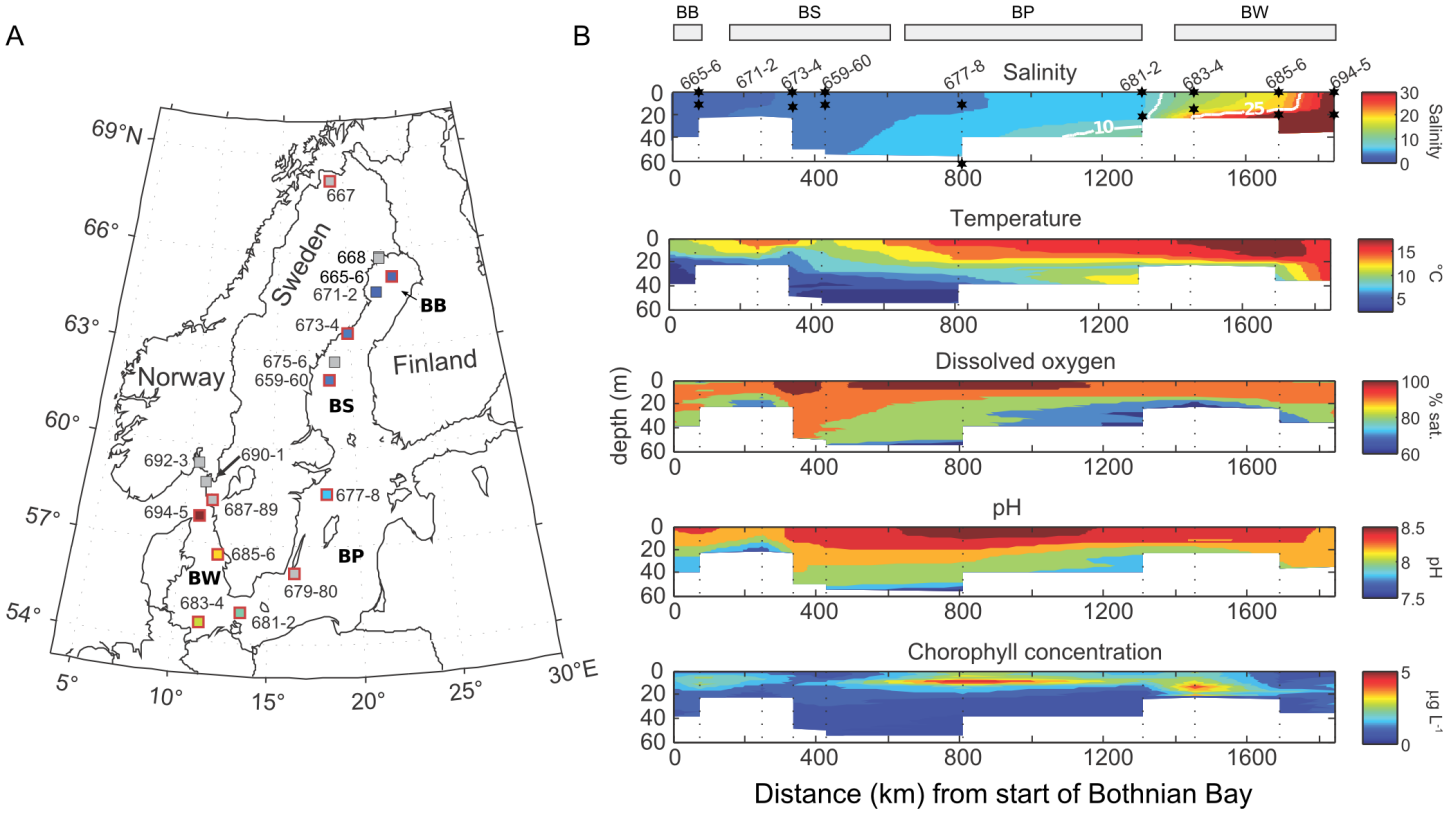
The Baltic Sea region and sampling locations. A) Squares with red outlines indicate sites with metagenomic sequencing. 16S rRNA gene tag sequencing was conducted at all sites. B) Temperature, salinity, pH, oxygen, and chlorophyll a measurements across the Baltic Sea transect. Only stations shown on the section were used for section interpolation. The transition from limnic to marine conditions along the 1800 km axis of the Baltic Sea leads to an extensive area of slowly varying brackish conditions. Suboxic, low-pH conditions exist throughout the central basin below 50–60 m. All environmental metadata is in table S1. Sub-basin abbreviations are marked on the map in bold font (BB – Bothnian Bay, BS – Bothnian Sea, BP – Baltic Proper, BW – Baltic West).

The Baltic Sea, one of the largest brackish ecosystems in the world (Fig. 1A), is intercontinental, non-tidal, and subject to constant freshwater input from major rivers in the north and saline water inflows from the North Sea resulting in a stable north-south salinity gradient of 2-10 practical salinity units (PSU) (Fig. 1B). The Baltic Sea is a ‘young’ water environment with the brackish state existing for about 4000 years. The low to moderate salinity levels are known to have imposed strong evolutionary constraints upon multicellular eukaryotes with limited species richness relative to adjacent fresh- and marine waters [15] while consequences for the microbial communities have remained largely unknown. The first large scale molecular (16S rRNA gene) survey of bacterial diversity in the Baltic Sea reported a community structured by salinity gradients with specific microbes at each salinity [16]. This contrasts with estuaries exhibiting faster turnover times and a mix of freshwater and marine bacteria at intermediate salinities [5]. Presumably, the long residence time in the Baltic Sea (3–30 years, [17]) allows for local adaptations along the salinity gradient. Thus the Baltic Sea provides an ideal natural model system for examining how salinity governs both microbial community composition and metabolic function. In an attempt to understand the evolutionary separation of freshwater and marine microbes we sequenced the genomes of microbial communities along the Baltic Sea salinity gradient and linked changes in functional potential to environmental parameters. Our results indicate that large-scale changes in core metabolic pathways determine which organisms succeed at different salinity regimes and that such profound changes explain the deep phylogenetic divide between freshwater and marine microbiomes.

## Materials and Methods

Samples were collected during the European leg of The Sorcerer II Global Ocean Sampling expedition in July 2009 with parallel environmental characterization using an integrated CTD-oxygen, chlorophyll a, and pH sensor package. For each country, a Marine Science Research Permit was submitted under the U.N. Conference on Law of the Sea to the U.S. State Department which contacted the Ministry of Foreign Affairs in the respective country. Submissions were then distributed to the appropriate agency in each country. For Sweden, permission was granted by the Swedish Coast Guard. For Denmark, permission was granted by the Danish Ministry of Foreign Affairs. For Germany, permission was granted by the German Federal Maritime and Hydrographic Agency (BSH). In addition, the Access and Benefit Sharing Focal Point was contacted under the U.N. Convention on Biological Diversity to ascertain whether additional permissions were required to access genetic resources. Separate applications were not required in the visited countries.

A total of 21 microbial communities were sampled at 11 locations within the brackish Baltic Sea area (Fig. 1A, Table S1). Eight locations were sampled at two depths (including seven surface/chl a maximum pairs and one chl a maximum/suboxic zone pair), one location was sampled at three depths (surface/chl a maximum/72 m) and at two locations only surface samples were taken. Environmental metadata and coordinates for each sampling location are given in Table S1. Sample preservation, extraction, and sequencing was conducted as described previously [3]. Reads were annotated using the JCVI metagenomic annotation pipeline [18], along with APIS [3], and fragment recruitment [2]. A global assembly of all sequence data was performed using the Newbler Assembler [19], with a final assembly of 490 million basepairs, a N50 of 1.5 Kbp, and a largest contig of 106 Kbp. Approximately 15,000 16S rRNA subunit amplicons were also sequenced for each location and size fraction and phylogenetically annotated as described previously [16]. All sequence and annotation data is available at CAMERA [20] under accession number CAM_P_0001109 and Metarep [21] (metarep.jcvi.org). Detailed descriptions of community composition determinations and regularized canonical correlation analysis (RCCA) are available in the Supplementary information.

## Results and Discussion

We collected aquatic samples from 21 freshwater, marine, and brackish sites within the Baltic Sea area, including the freshwater arctic lake Torne Träsk. The transect spans the Bothnian Bay, Bothnian Sea and the large Baltic Proper sub-basins, in turn semi-connected to the marine waters of the Swedish west coast, here referred to as Baltic West (Fig. 1A). Interpolated depth profiles of temperature, oxygen, salinity, pH, and chlorophyll a fluorescence (chl a) place the samples in context of a basin wide salinity gradient (Fig. 1B). The majority of the samples were from the relatively young (<30 years) surface layer of the Baltic Sea. Two samples were taken below the halocline and thus capture older waters: GS678 represents an oxygen minimum sample taken at 74 m within the Baltic Proper and GS689 at 72 m depth in the oxygenated waters in fjord Gullmarn on the Swedish west coast. Comparisons with the long-term Baltic Sea monitoring program verified that the conditions were typical for the sampled locations at that time of year (Fig. S1). Samples were fractionated into particle size classes of 0.1–0.8, 0.8–3.0 and 3.0–200 µm, each of which was shotgun-sequenced using pyrosequencing. The dataset comprises 7.6 billion nucleotides in 20.9 million reads (avg. length = 324 bp), with 22.9 million translated amino acid sequences (Table S2). In addition, approximately 15,000 16S rRNA subunit sequences were obtained for each size-fractionated sample.

**Figure 2:**
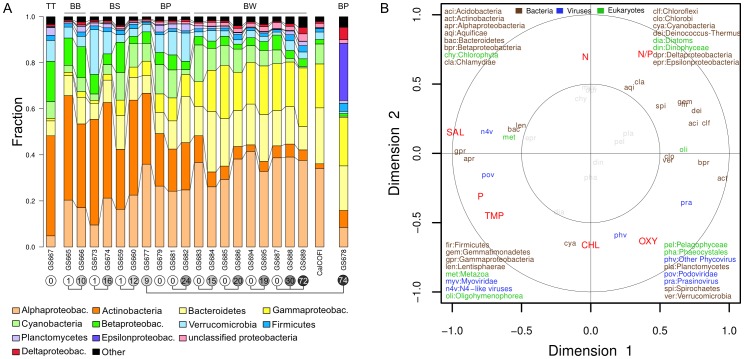
Salinity-driven shifts in bacterial community composition. A) Bacterial communities characterized by AMPHORA2. The 12 most abundant groups are shown with “Other” defined as the remaining taxonomic groups. Sequences have been combined for all non-viral size fractions (0.1, 0.8 and 3.0 µm) with normalization for genome equivalents. Sampling depth (m) is indicated within circles below sample names, with interconnecting circles indicating samples from the same location. The corresponding basin for samples is shown in the top margin: TT = Torne Träsk, BB = Bothnian Bay, BS = Bothnian Sea, BP = Baltic Proper, BW = Baltic West. A merged set of metagenomes from the coastal eastern Pacific (CalCOFI) was included for comparison. B) RCCA analysis of community composition. The biplot shows correlations between environmental (red) and phylogenetic (color coded by superkingdom) variables with the corresponding first and second canonical variates. The first two canonical variates explain 65% of the environmental variables and 49% of the organismal variables. Biplots for a RCCA at the genus level is shown in figure S5. The outer circle (1) and inner circles (0.5) display the amount of variance explained by the linear combinations of variables. The variance explained for points within the inner circle is less than 25%, thus these have been dimmed.

Microbial community composition in the Baltic Sea varied dramatically relative to the considerably more geographically distant ocean sites in previous Global Ocean Sampling (GOS) datasets, both in terms of magnitude and taxonomic hierarchy [2], [4], [22]. Specifically, the most abundant bacterial phyla and classes changed in a continuous fashion along the transect (Fig. 2A) while classes of viruses and phytoplankton divisions were equally dynamic but exhibited a more stochastic pattern (Fig. S2). These trends were robust to annotation biases as 16S rRNA gene amplicon sequencing, the AMPHORA2 classification system [23] (31 core genes) and automated phylogenetic inference system (APIS [3], all genes) showed strong correlations (Fig. S3). Examining the individual size fractionated metagenomes revealed that the changes in bacterial community composition occur almost entirely in the 0.1–0.8 and 0.8–3.0 µm size fractions, which likely contain free-living bacteria, and not the 3–200 µm size fraction (Fig. S4), despite the salinity being equal. Bacteria found in these larger size fractions have substantially larger average genome sizes relative to those found in the smaller size fractions (see Table S3 and [3]), which allows for an expanded regulatory and metabolic capacity [24]. We interpret our results to suggest that expanded metabolic and regulatory potential relax the influence of environment on genomic contents.

In order to investigate the environmental effect on the microbial community composition we used regularized canonical correlation analysis (RCCA), a multivariate method that analyzes the correlation between sets of several independent (e.g. environmental data) and dependent (e.g. metagenomic signatures) variables [25], [26]. Briefly, it calculates linear combinations of variables (canonical variates) in each set and finds the maximum (canonical) correlation between the combinations. It simultaneously measures the relationship between and within the variable sets and weights the contribution of the variables in each set to the global relationship. This makes the method robust to dependencies among the variables. RCCA of our environmental and metagenomic data showed that the first linear combination of environmental variables (canonical variate) was primarily and negatively associated with salinity (r = −0.976) at the phylum level (Fig. 2B) for combined size fractions, with similar results at the genus level (Fig. S5). Thus, the changes in bacterial community composition were primarily driven by changes in salinity. Organismal phyla/classes preferentially found in low-salinity habitats were actinobacteria, chloroflexi, betaproteobacteria, and the eukaryote ciliate oligohymenophora. In contrast, gammaproteobacteria, alphaproteobacteria, bacteroidetes, and N4-like viruses (*Podoviridae*) were positively associated with higher salinity (Fig. 2B). The second highest canonical variate (canonical correlation 0.734) associated positively with the N∶P nutrient ratio and negatively with chl a and oxygen. Organisms mostly associated with this environmental combination were aquificae, cyanobacteria, and phycoviridae. These results recapitulate the trends observed for single or multiple classes of bacteria in previous surveys [5]–[7], [9], [10], [16], while adding new insight to viruses and eukaryotes. Our experimental design also allowed us to relate these distribution patterns to potential patterns in functional genes, which was not done in the previous studies. Due to the general lack of statistically significant dependence of eukaryotes and viruses on measured environmental variables we focused the functional analysis on bacteria. We also note that bacterial reads comprised 80.1% of the complete dataset and thus provide the coverage necessary for detailed within taxonomy analyses not possible with viruses or eukaryotes.

**Figure 3:**
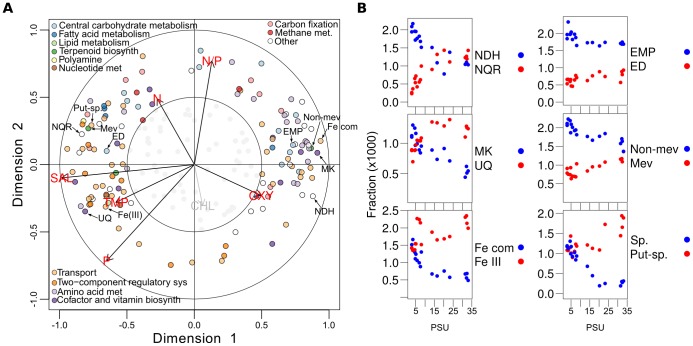
Salinity and functional potential. A) KEGG module and metadata correlations with first and second canonical variates for the bacterial communities in combined size fractions. Text labels and associated arrows indicate the position of analogous modules shown in panel B. The outer circle (1) and inner circles (0.5) display the amount of variance explained by the linear combinations of variables. The variance explained for points within the inner circle is less than 25%, thus these have been dimmed. Biplots for each size fraction and the combined communities are shown in fig. S6. B) Plots showing the distribution of analogous central metabolic pathways highlighted in panel A along the salinity gradient. Note that the spermidine transport system (‘Sp.’) is not shown in panel A due to its non-normal distribution.

A RCCA analysis of environmental variables and KEGG functional modules of bacteria, including metabolic pathways, transporters, and two-component transcriptional regulation systems showed that salinity was the primary constituent of the first environmental canonical variate M1 (r = −0.986) while M2 correlated positively with N∶P ratio (r = 0.770) and negatively with total phosphorous (r = −0.716). The linear combination of modules that maximized correlation with M1 (canonical correlation 0.997) separated a majority of individual KEGG modules (Fig. 3A). This analysis shows that, as was the case for community composition, salinity was the main determinant of nearly all large-scale differences in the metabolic potential of the Baltic Sea bacterial communities. Importantly, these results show correlations between combinations of variables (environmental and metagenomic), thereby minimizing confounding effects potentially introduced by co-variation between salinity and other environmental variables. Similar M1 or salinity-driven KEGG module correlations were observed for both 0.1 and 0.8 µm size fractions (Fig. S6A–B), while bacteria in the largest size fraction (3.0–200 µm), with larger genomes and perhaps attached to particles or aggregates, displayed slightly different profiles, including a better correlation with oxygen and chl a (Fig. S6C–D). This latter result is consistent with the general lack of a salinity-dependent biogeography for the largest size fraction noted above (Fig. S4). Thus there appears to be a scenario where increased genome size, potentially facilitated by a higher trophic status, overrules the effects of salinity observed in pelagic bacteria. Overall, these results are highly different from previous RCCA analysis of surface marine metagenomes, all collected from sites with PSU values greater than 30, where nitrate and temperature were more important factors in governing community structure [22]. Notably, our use of KEGG modules allows for an explicit exploration of linked pathways and metabolic changes.

Strikingly, we found that salinity strongly influenced the usage of central analogous metabolic pathways, each of which shares products or substrates with another salinity-influenced pathway (Fig. 3, Fig. S7). Analogous metabolic pathways achieve approximately the same result, although using different intermediate metabolites, energetics, and genes. For instance, the H^+^-translocating (NDH) and the Na^+^-translocating (NQR) NADH:quinone dehydrogenases, key enzymes in bacterial respiration, were more abundant in low and high salinity environments, respectively (Fig. 3B, Fig. S7). Biosynthetic pathways for two major isoprenoid quinones, menaquinone (MK) and ubiquinone (UQ) were also found to correlate with low and high salinity environments, respectively. The NQR dehydrogenase effectively exports sodium from the cell and has been identified in a limited number of cultivable marine bacteria [27]. In addition, the energetics of the NQR dehydrogenase makes it dependent on the high-potential UQ electron acceptor [28], as reflected in our results (Fig. 3). NDH dehydrogenases generate a proton motive force across the cytoplasmic membrane (i.e. 1 1/3 more ATP per quinone reduction) and use the low-potential MK electron acceptor. Consequently, both the NQR and UQ pathways dominate at higher salinities, while NDH and MK pathways at low salinity. Similar trends were observed for the two non-overlapping biochemical modules for glycolysis, the Embden-Meyerhof (EMP) and Entner-Doudoroff (ED) pathways, and the non-mevalonate and mevalonate biosynthetic pathways used for isoprenoid biosynthesis (Fig. 3, Fig. S7). The pathways of glycolysis and isoprenoid biosynthesis generate the basic building blocks required for the two quinone biosynthesis pathways, though the explicit linkages noted here are novel (Fig. S7). Overall, these salinity-associated differences were found in metabolic pathways that are interlinked by substrates within each salinity regime. Consistent with changes in glycolytic pathways observed here, a recent comparison of marine and freshwater metagenomes showed significant overrepresentation of the EMP enzyme 6-phosphofructokinase in freshwater metagenomes [13].

While Figure 3B presents the abundance of pathways along the entire salinity gradient, some pathways are selected for in specific salinity ranges. For instance, changes in dehydrogenase utilization and use of glycolytic pathways were most evident at salinity levels below 15 PSU, while quinone and isoprenoid biosynthesis changed in a linear fashion across the entire salinity gradient. These results provide insight to the distribution of isoprenoid and glycolysis pathways in natural and predominantly uncultivated microbes across salinity gradients, which was recently reviewed for cultivated organisms without regard to environmental parameters [29], [30]. The metabolic units we associated with high salinity (e.g. UQ quinones and ED glycolysis) and low salinity (e.g. MK quinones and EMP glycolysis) have been linked to aerobic and anaerobic strategies, respectively, in cultivated prokaryotes [30], [31]. It has been suggested that this differential usage of quinones is due to their respective midpoint potentials, but examples of MK usage despite unfavorable redox potential have been reported [31]. Furthermore, while there is strong evidence that cultivated obligate anaerobes almost exclusively rely on the EMP pathway there is great variability in the choice of glycolytic pathway in cultivated facultative and obligate aerobes [30] that will be dominant in our oxic sample set. Our data indicate a weak negative relationship between oxygen and salinity (Fig. 3A) and it is thus unlikely that the increased usage of the EMP pathway in low-salinity environments is due to a higher proportion of anaerobic prokaryotes. The preferential usage of the EMP pathway, which yields more ATP compared to the ED analog, and the NDH dehydrogenase, which is used in ATP-producing oxidative phosphorylation, in low-salinity environments (Fig. 3) instead indicate a reliance on these pathways for ATP-production in freshwater bacteria. Potentially, marine bacteria rely to a greater extent on other sources of ATP, such as proteorhodopsins. Taken together, our cultivation-independent analysis shows that salinity is the main driver of changes related to respiratory and glycolytic pathways in the naturally occurring bacterial populations.

Some modules lacking analogous pathways were found preferentially in marine or freshwater samples, including the *com* system of competence-related DNA uptake (freshwater) and glutathione biosynthesis (marine). The increased abundance of genes for DNA uptake in freshwater samples indicates a higher level of gene-sharing between bacteria in these environments, or an increased use of naked DNA as a source of nutrients. In addition, surface-attached microbial communities have been shown to be enriched in gene-exchange functions [32] and the higher particle concentration often associated with brackish and low-salinity estuarine waters may thus facilitate cell-to-cell interactions and DNA exchange opportunities. Moreover, several ABC transporter systems with similar substrates, including those for Fe-complex (low salinity) vs. Fe (III) (high salinity) and for the polyamines spermidine (low salinity) and putrescine (high salinity), also exhibited salinity dependent abundances (Fig. S8). Not unexpectedly, import systems for the osmoprotectants glycine betaine (GBT) and choline, and lipoprotein exporters were enriched in marine sites with the converse scenario for the FtsX/FtsE putative transporter previously implicated in salinity tolerance [33]. Further, sodium export and zinc transport systems showed a strong correlation with salinity, corroborating recent findings from comparisons of freshwater and marine metagenomes [13]. However, our results show that transporters for osmolytes exhibited the greatest increase in abundance at levels greater than 20 PSU, thereby offering clarity into adaptations across continuous changes in salinity.

To some extent, phylogeny and functional potential are interlinked and one possible explanation for some of the results invokes salinity selecting for different bacterial phyla or classes for reasons beyond the metabolic changes. Glutathione biosynthesis provides a clear example of this; actinobacteria have replaced glutathione biosynthesis with mycothiol synthesis, and the decreased abundance of glutathione biosynthesis pathways at low salinities mirrors phylogenetic choice. Collating the phylogenetic and KEGG annotations prior to RCCA analyses on phylogenetic subgroups, we found salinity to be a major determinant of functional variations within the nine most abundant organismal groups that have enough coverage for statistical analyses (Fig. S9). In four of these nine groups (actinobacteria, bacteroidetes, cyanobacteria and verrucomicrobia), salinity was the primary constituent of the first environmental variate, while for three additional groups (betaproteobacteria, firmicutes and gammaproteobacteria) salinity was the secondary constituent. For instance, the Fe (III) and Fe-complex transporters were correlated with salinity for the actinobacteria, bacteroidetes and firmicutes. Non-mevalonate isoprenoid biosynthesis and the EMP glycolytic pathway were preferred at low salinity in the firmicutes, verrucomicrobia, actinobacteria and bacteroidetes with shifts towards the ED pathway at high salinity for the latter two groups. We also found preferential usage of the MK synthesis pathway at low salinity for the gammaproteobacteria and finally an increased abundance of the H^+^ translocating dehydrogenase at lower salinity in the actinobacteria, betaproteobacteria, gammaproteobacteria, verrucomicrobia and bacteroidetes with a strong shift towards the Na^+^ translocating enzyme at higher salinity in the latter. Many of these sub-phyla metabolic changes represent deviations from the predominant metabolic repertoire of each individual phyla based on genomic surveys [29], [30]. While not all changes in central analogous pathways were observed in all bacteria, we stress that salinity was a major determinant of variations in function, both at the community level (Fig. 3) and within the most abundant bacterial phyla and classes (Fig. S9).

**Figure 4:**
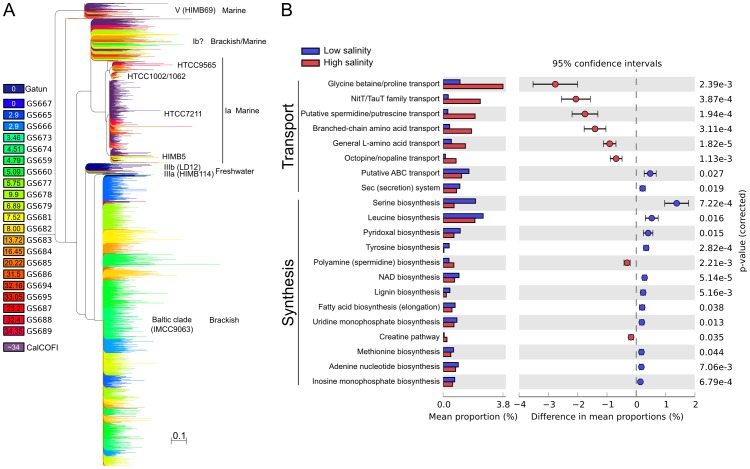
Baltic Sea SAR11 phylogeny and function. A) Metagenomic sequences classified as SAR11 placed onto a reference tree built from 31 concatenated core genes (see Methods). The Rickettsiales sister group (not shown for clarity) of *Candidatus* Pelagibacter was chosen as outgroup from AMPHORA2 reference trees. Terminal branches are colored according to the legend in the left margin. Sequences with likelihood weights<0.9 were pruned from the tree. Branches corresponding to sequences from the freshwater Lake Gatun and coastal California metagenomes are colored purple and dark blue, respectively. Numbers in the boxes indicate salinity (PSU). B) Functional differences between the low and high salinity SAR11 populations. Plot of differences in KEGG categories related to biosynthesis (“Synthesis”) and transport (“Transport”) of amino acids, nucleotides and cofactors. Bar plots show mean proportions for each category in the different populations and the confidence interval. Welch's t-test p-values (corrected using the Bonferroni method) are shown in the right margin of the plot.

To further examine environmental influence on function within a taxa, we examined the phylogenetic distribution and functional potential of the most abundant order in our dataset, the alphaproteobacteria of the SAR11 cluster (order *Pelagibacterales* [34]), found across the whole Baltic Sea transect (Fig. S10A). The most abundant bacterial order in open oceans [35], *Pelagibacterales* comprise several identified subgroups (ecotypes) [34], [36]. Most SAR11 sequences from the low salinity (<10 PSU) Baltic Sea samples clustered with clade IIIa *Ca*. Pelagibacter IMCC9063 (isolated from brackish waters, Svalbard [37]) and to a lesser degree with HIMB114 (Fig. 4A). Lake Gatun SAR11 sequences formed a freshwater sister group, LD12 or IIIb, while sequences from the coastal Pacific and the marine Baltic West clustered within the marine SAR11 Ia sub-clade. Sequences from mid to high salinity environments in the Baltic Sea formed a sister group to Ia, potentially Ib, a sub-clade without a reference genome. Numerous analyses indicate the Baltic populations as unique species from currently cultivated strains. For example, reads from low salinity sites were aligned to the IMCC9063 genome at 70–80% nucleotide identity (Fig. S10B), consistent with it being a new species (generally 75% average nucleotide identity). Further, the phylogenetic placement (Fig. 4A) shows a high amino-acid diversity of individual core proteins. This whole-genome and multiple protein analysis of SAR11 corroborated and expanded upon previous biogeographical findings from 16S rRNA gene based analyses [7], and illustrated the existence of novel species within the freshwater/brackish SAR11 clades.

Organisms in the order *Pelagibacterales* have small genomes and share a large core genome [34], indicating that variations in the pan-genome of SAR11 would explain its salinity-associated distribution. We therefore analysed variations in functional potential within the dataset for the reads and assembled contigs classified as *Pelagibacterales*, an analysis facilitated by the wealth of *Pelagibacterales* genomes. The SAR11 assembly pool consisted of 20,309 contigs with an N50 length of 1,730 bp and a maximum length of 28,089 bp. Large differences in functional potential were evident for SAR11 populations along the Baltic Sea salinity gradient (Fig. 4B, Fig. S10C). The data showed different strategies for amino acid, nucleotide and cofactor utilization, as well as for osmolyte synthesis between the low- and high-salinity SAR11 populations, the former adapted to *de novo* synthesis and the latter to uptake of these compounds from the surrounding medium (e.g. synthesis vs. uptake of glycine; Fig. 4B). An overrepresentation of amino-acid import systems was recently reported in high- compared to low-salinity environments [13] and our data suggest that these differences may be driven by large SAR11 populations with contrasting metabolic strategies. The mevalonate and non-mevalonate pathways for isoprenoid biosynthesis were positively and negatively correlated with salinity, respectively, in the read-based SAR11 analysis, mirroring the community level pattern (Fig. 3, Fig. S7). In addition, SAR11 transport systems for polyamines, GBT and Fe (III) were positively correlated with salinity. An assembly-driven analysis corroborated the read-based results, and also highlighted differences in ascorbate biosynthesis pathways between the low and high-salinity populations (Fig. S10C). Glycolysis, a trait completely lost in some SAR11 strains [38], via both the EMP and ED pathways were negatively correlated with salinity in the Baltic Sea dataset (R = −0.89 and R = −0.87, p<2.2e^−16^). In contrast to other proteobacteria, *Pelagibacterales* did not change their preference for NAD dehydrogenase or quinones. The correlation between type I dehydrogenase and salinity was not statistically significant within the *Pelagibacterales* and the NQR type was missing altogether. This analysis of functional potential within the SAR11 lineage shows that numerous pathways are differentially abundant at the varying salinity regimes. Essentially, the low-salinity Baltic Sea populations of SAR11 have acquired multiple energy generating catabolic pathways not found in marine SAR11 populations (Fig. 4B, Fig. S10). Overall, the phylogenetic shifts in SAR11 populations (Fig. 4A) are underpinned by large-scale metabolic changes, and this genetic flexibility has likely helped SAR11 overcome salinity gradients and contributed to its ecological success.

## Summary

This dataset differs from previous metagenomic surveys examining salinity in its resolution of a continuous gradient, consistent wet-lab and informatics techniques, which is important for comparative genomics [14], its focus on the most geographically prevalent salinity gradient, that is the range between freshwater and marine systems, and a multivariate statistical analysis that reveals systematic restructuring of the successful bacterial central metabolism across this gradient.

The metabolic pathways we identified as “salinity influenced” are primordial parts of bacterial metabolism and require a considerable set of different genes, which offers a plausible explanation for the deep phylogenetic separation between the organisms that dominate at the various salinity regimes. We propose that metabolic adaptations dictate which bacteria succeed at a given salinity. Moreover, the use of pathways with a higher ATP-yield (i.e. a proton versus sodium pumping complex I and the EMP versus the ED glycolytic pathway) at low salinity could impact bacterial growth efficiency, i.e. the amount of carbon assimilated into biomass relative to that released as CO_2_. Growth efficiencies vary from 0.05–0.5 and help determine if an aquatic environment is either a sink or source of CO_2_ to the atmosphere [39], [40]. Previous surveys across salinity gradients have shown conflicting results in the relationship between salinity and bacterial respiration [40]–[43] and our findings stress the need for further work to determine to what extent salinity influences CO_2_ assimilation. This will help ascertain whether salinity should be incorporated in future climate change models that currently predict an increase in precipitation and subsequent reduction in salinity levels [44], [45].

## Supporting Information

Figure S1 **Seasonal variations in metadata variables at or near sites along the Baltic Sea transect.** Seasonal measurements were acquired from the SMHI and HELCOM annual archives with values from December 1898 to January 2012. The closest set of monitoring stations were selected for each site in the transect (limited to a maximum distance of 5–30 km and maximum sampling depth difference of 10 m) and values were averaged for these stations. The x-axis corresponds to one year (Jan–Dec) and the blue line shows the average of each month. Standard deviation is indicated by the yellow area around each line. Number of total measurements for each month is shown in brackets on the x-axis. The measured data during sampling (June/July 2009) is shown as a red circle. Since no monitoring data was available for Torne Träsk (GS667) only the data measured during the 2009 sampling is shown (left). For the rest of the sampling sites, a plot shaded in red indicates that no adjacent monitoring data was present. The corresponding Baltic Sea sub-basin for each set of samples is shown above the figure: TT  =  Torne Träsk, BB  =  Bothnian Bay, BS  =  Bothnian Sea, BP  =  Baltic Proper, BW  =  Baltic West.
(PDF)

Figure S2 **Taxonomic composition of eukaryotes.** (A) and virus (B) along the Baltic Sea transect. All data based on APIS. Samples are arranged from north (low-salinity) to south (high-salinity) and the corresponding Baltic Sea sub-basin for each set of samples is shown below each panel: BB  =  Bothnian Bay, BS  =  Bothnian Sea, BP  =  Baltic Proper, BW  =  Baltic West.
(PDF)

Figure S3 **Correlation plots of bacterial taxonomic composition as assayed using AMPHORA, APIS and 16S rRNA gene amplicon sequencing analysis.**
(PDF)

Figure S4 **Taxonomic composition of bacteria in the A) 0.1–0.8 µm, B) 0.8–3.0 µm, and C) 3.0–200 µm size fractions at each site.** The same bacterial phyla/classes as in Figure 2A are shown. Sampling depth (m) is indicated within circles below sample names, with interconnecting circles indicating samples from the same location. The corresponding basin for samples is shown in the top margin: TT = Torne Träsk, BB = Bothnian Bay, BS = Bothnian Sea, BP = Baltic Proper, BW = Baltic West. CalCOFI represents samples taken from the coastal eastern Pacific.
(PDF)

Figure S5 **Regularized canonical correlation analysis of the abundance of bacterial phylotypes at the genus level.** For ease of visualization, each genus is color coded according to Phylum. The analysis recapitulates many of the trends from the Phyla level analysis, with actinobacteria, verrucamicrobia, and many bacteroidetes being found at low salinity, while gamma and alpha proteobacterial are found at high. This analysis does provide more detailed information, such as the alphaproteobacteria in the SAR11 lineage, HIMB114, being found at low salinity that is recapitulated by other analyses (Fig. 5A).
(PDF)

Figure S6 **A) Correlation of functional modules against first variant M1 for both 0.1 and 0.8 um communities.** B) A sample by sample comparison of the 1st dimension of 0.1 (x axis) and 0.8 (y axis) fractions. C) Module and metadata correlations with first and second canonical variates for the bacterial communities found in the 3.0–200 µm fraction. D) A sample by sample comparison of the 1st dimension of 0.8 and 3.0 fractions.
(PDF)

Figure S7 **A schematic of community scale metabolic adaptations across salinity gradients.** Note that while the metabolic pathways are not expressly linked, they share substrates and products and thus have the potential.
(PDF)

Figure S8 **Distribution of prokaryotic-type ABC transporters in the Baltic dataset (see Methods for details).** Heatmap color scaling is based on rows. *“Putative salt transporter” has been suggested to be involved in salt transport and/or cell division.
(PDF)

Figure S9 **Regularized canonical correlation analysis of functional modules within the 9 most abundant bacterial phyla or classes in the total dataset.** For each plot, the outer circle (1) and inner circles (0.5) display the amount of variance explained by the linear combinations of variables. The variance explained for points within the inner circle is less than 25%, thus these have been dimmed. Labelled arrows show the correlation coefficient of environmental variables. Each point represents a KEGG functional module and is colored according to its parent metabolic category (see KEGG Metabolic Category legend). Specific modules discussed in the text are indicated in each plot by numbers (1–13) and described in the KEGG Module legend with colored circles matching their parent metabolic category.
(PDF)

Figure S10 **A) Annotations of SAR11 by APIS. SAR11 (Pelagibacterales) are found across the entire transect, but in a species and clade specific manner.** Clade IIIa (IMCC9063) is primarily found in the low salinity environments while clade Ia (Can. Pelagibacter ubique and HTCC7211) dominates the high salinity environment. B) Fragment recruitment plots of different Pelagibacterales genomes. While IMCC9063 is the most similar to those found in the low salinity waters (cold colors), the population in this region is only 70–80% average nucleotide identity and should be considered a distinct species and possibly sub-clade. C) Frequency of functional modules related to transport and biosynthesis in the SAR11-like combined assembly. Contigs were divided into brackish and marine bins based on read proportion from samples in the different environments (brackish: GS665-GS682, marine: GS683-GS695).
(PDF)

Table S1 **Sites and environmental data for the sites where metagenomic sequencing was conducted.**
(DOC)

Table S2 **Number of peptides generated from pyrosequencing reads for each filter at each site, along with the portion that could be annotated using the JCVI metagenomic annotation pipeline.**
(DOC)

Table S3 **Bacterial genome size estimates for samples.**
(XLS)

Materials and Methods S1 **Detailed description of materials and methods.**
(DOC)
