# Supplementary figures and images for: Functional Tradeoffs Underpin Salinity-Driven Divergence in Microbial Community Composition
Source: PLoS One. 2014 Feb 27;9(2):e89549. doi: 10.1371/journal.pone.0089549 (PMC3937345; doi:10.1371/journal.pone.0089549)

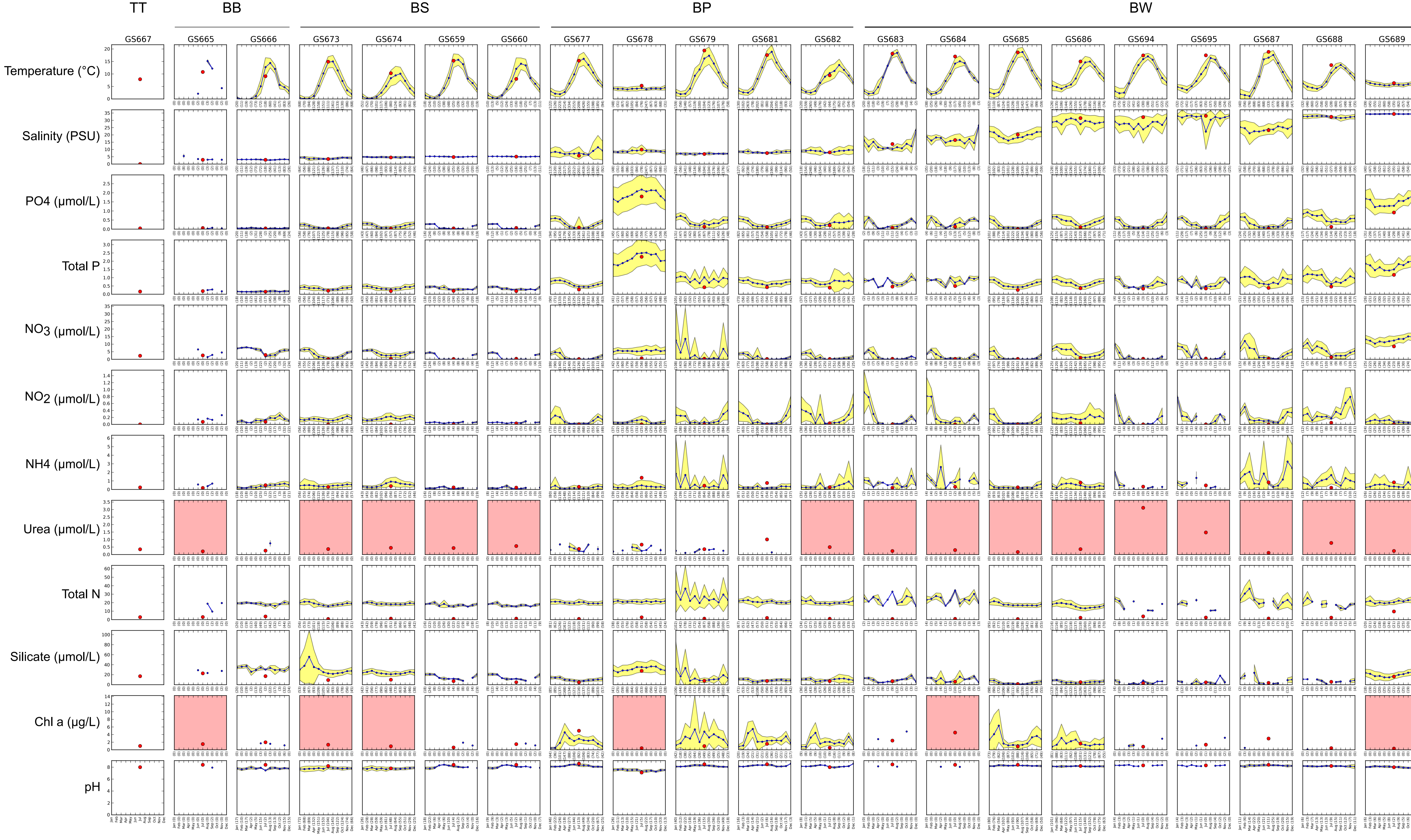

Supplement: Figure S1 — Seasonal variations in metadata variables at or near sites along the Baltic Sea transect. Seasonal measurements were acquired from the SMHI and HELCOM annual archives with values from December 1898 to January 2012. The closest set of monitoring stations were selected for each site in the transect (limited to a maximum distance of 5–30 km and maximum sampling depth difference of 10 m) and values were averaged for these stations. The x-axis corresponds to one year (Jan–Dec) and the blue line shows the average of each month. Standard deviation is indicated by the yellow area around each line. Number of total measurements for each month is shown in brackets on the x-axis. The measured data during sampling (June/July 2009) is shown as a red circle. Since no monitoring data was available for Torne Träsk (GS667) only the data measured during the 2009 sampling is shown (left). For the rest of the sampling sites, a plot shaded in red indicates that no adjacent monitoring data was present. The corresponding Baltic Sea sub-basin for each set of samples is shown above the figure: TT = Torne Träsk, BB = Bothnian Bay, BS = Bothnian Sea, BP = Baltic Proper, BW = Baltic West. (PDF) [file pone.0089549.s001.pdf]

A

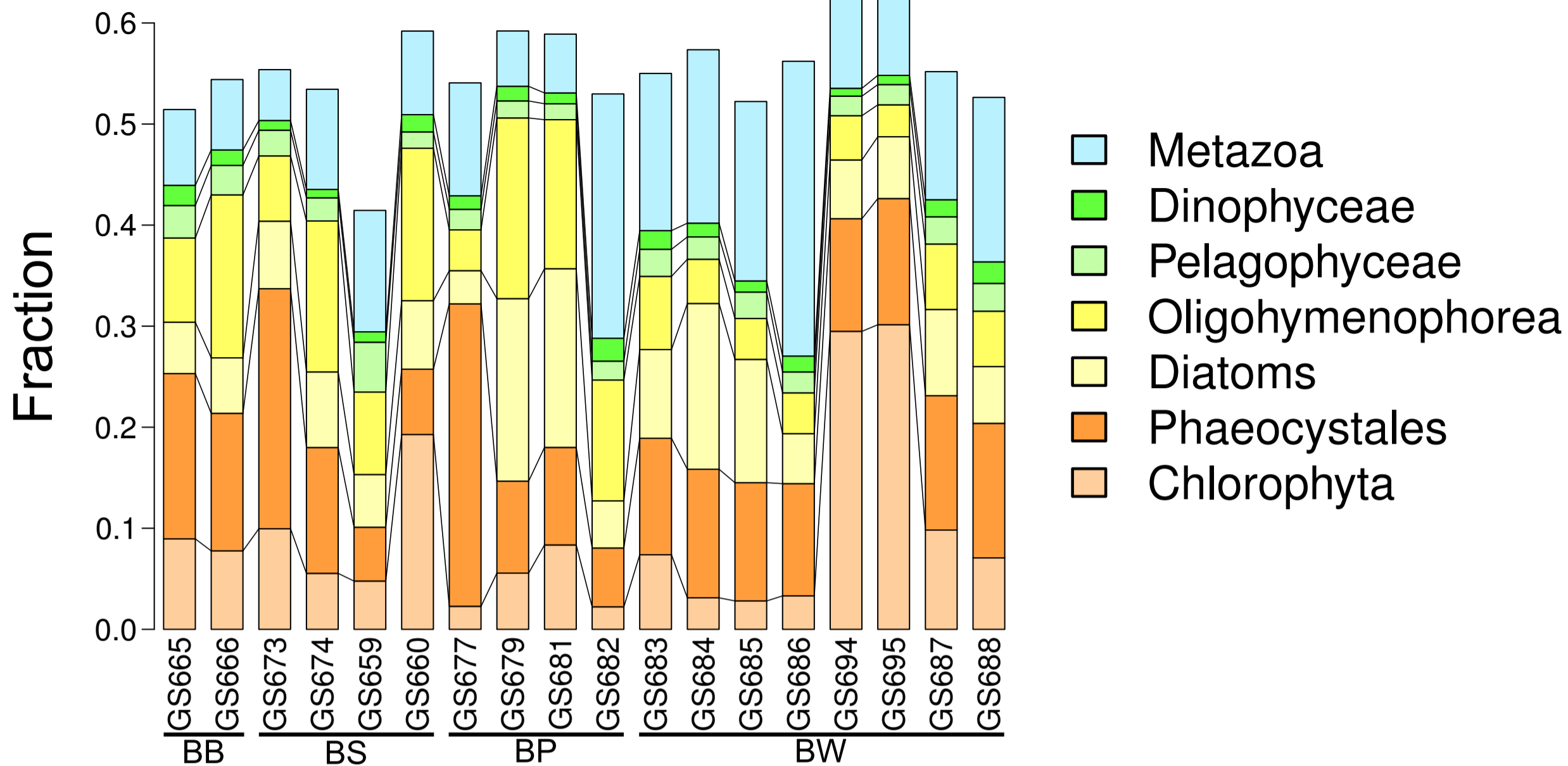

B

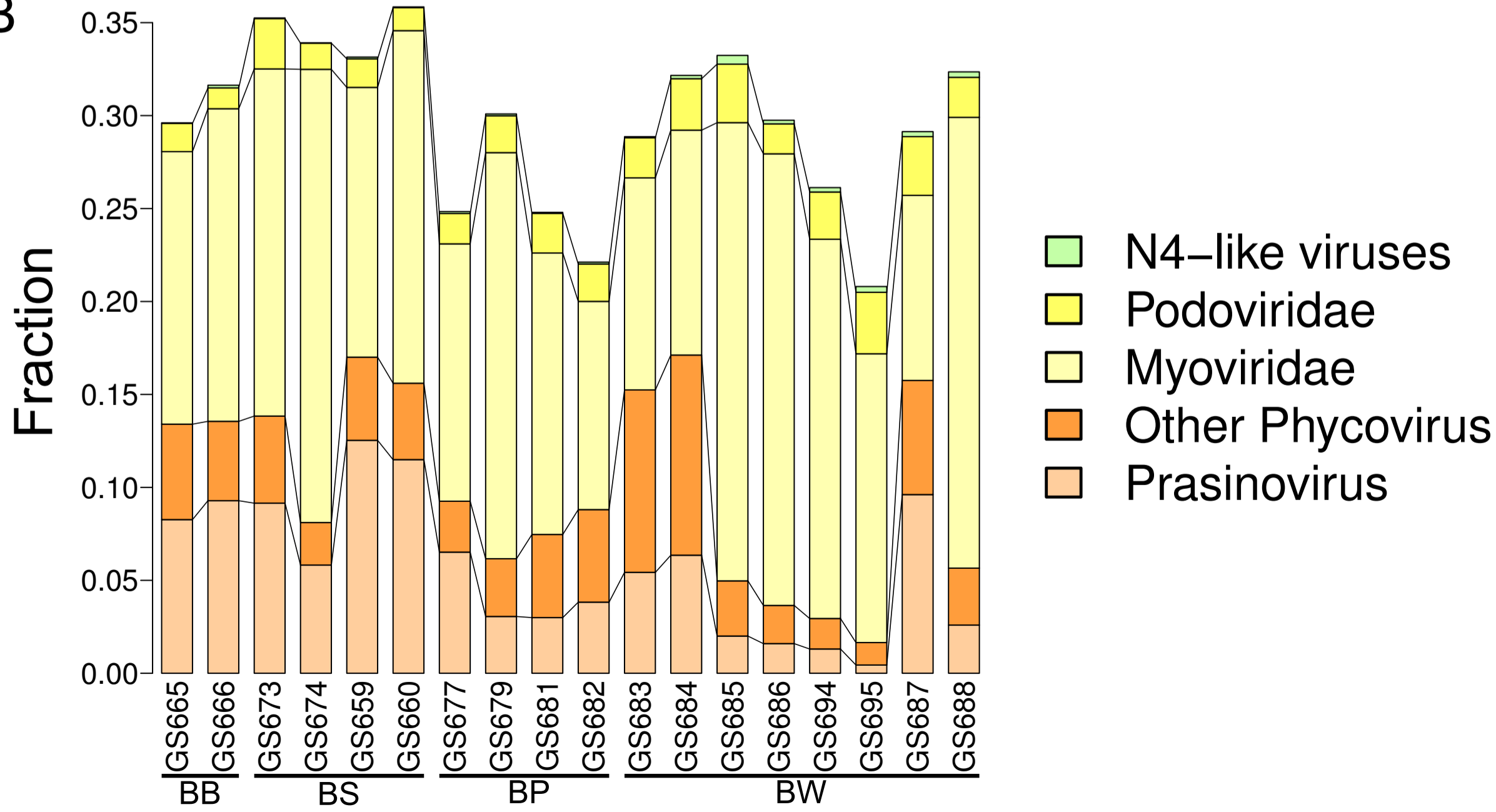

Supplement: Figure S2 — Taxonomic composition of eukaryotes. (A) and virus (B) along the Baltic Sea transect. All data based on APIS. Samples are arranged from north (low-salinity) to south (high-salinity) and the corresponding Baltic Sea sub-basin for each set of samples is shown below each panel: BB = Bothnian Bay, BS = Bothnian Sea, BP = Baltic Proper, BW = Baltic West. (PDF) [file pone.0089549.s002.pdf]

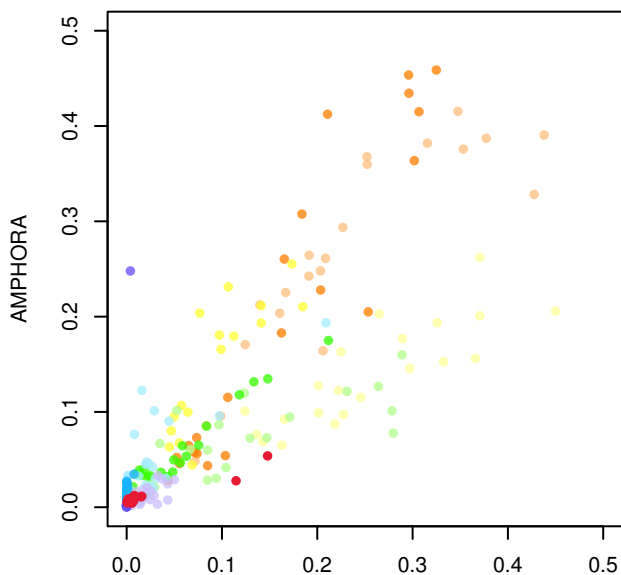

Amplicon  
 $R = 0.863$   $p = 0.00063$

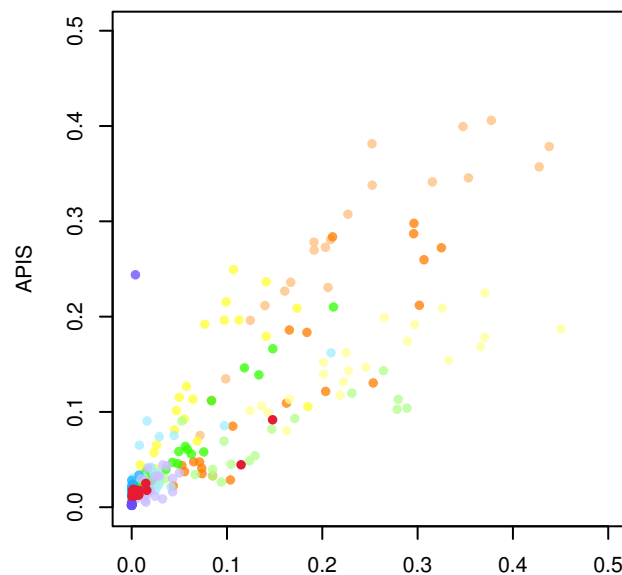

Amplicon  
 $R = 0.88$   $p = 0.00035$

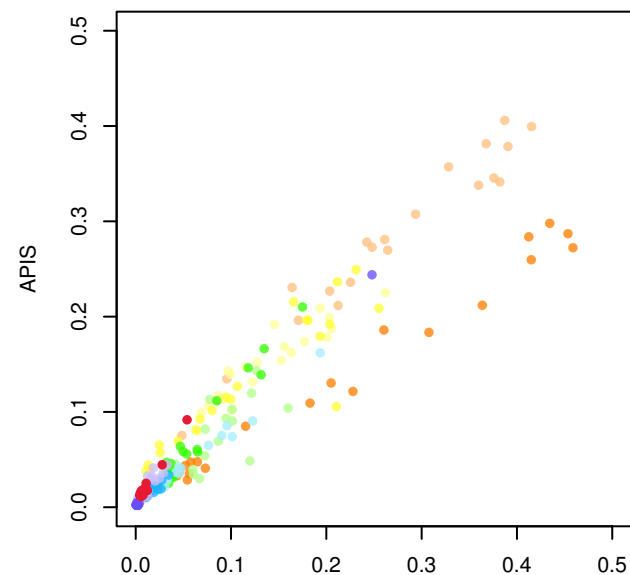

AMPHORA  
 $R = 0.918$   $p = 7e-05$

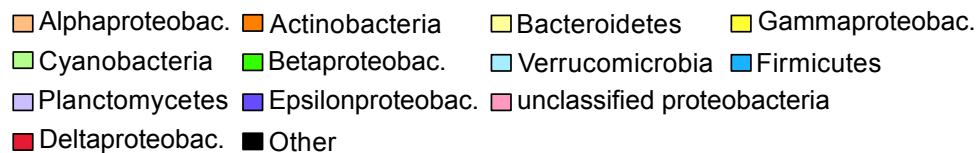

Supplement: Figure S3 — Correlation plots of bacterial taxonomic composition as assayed using AMPHORA, APIS and 16S rRNA gene amplicon sequencing analysis. (PDF) [file pone.0089549.s003.pdf]

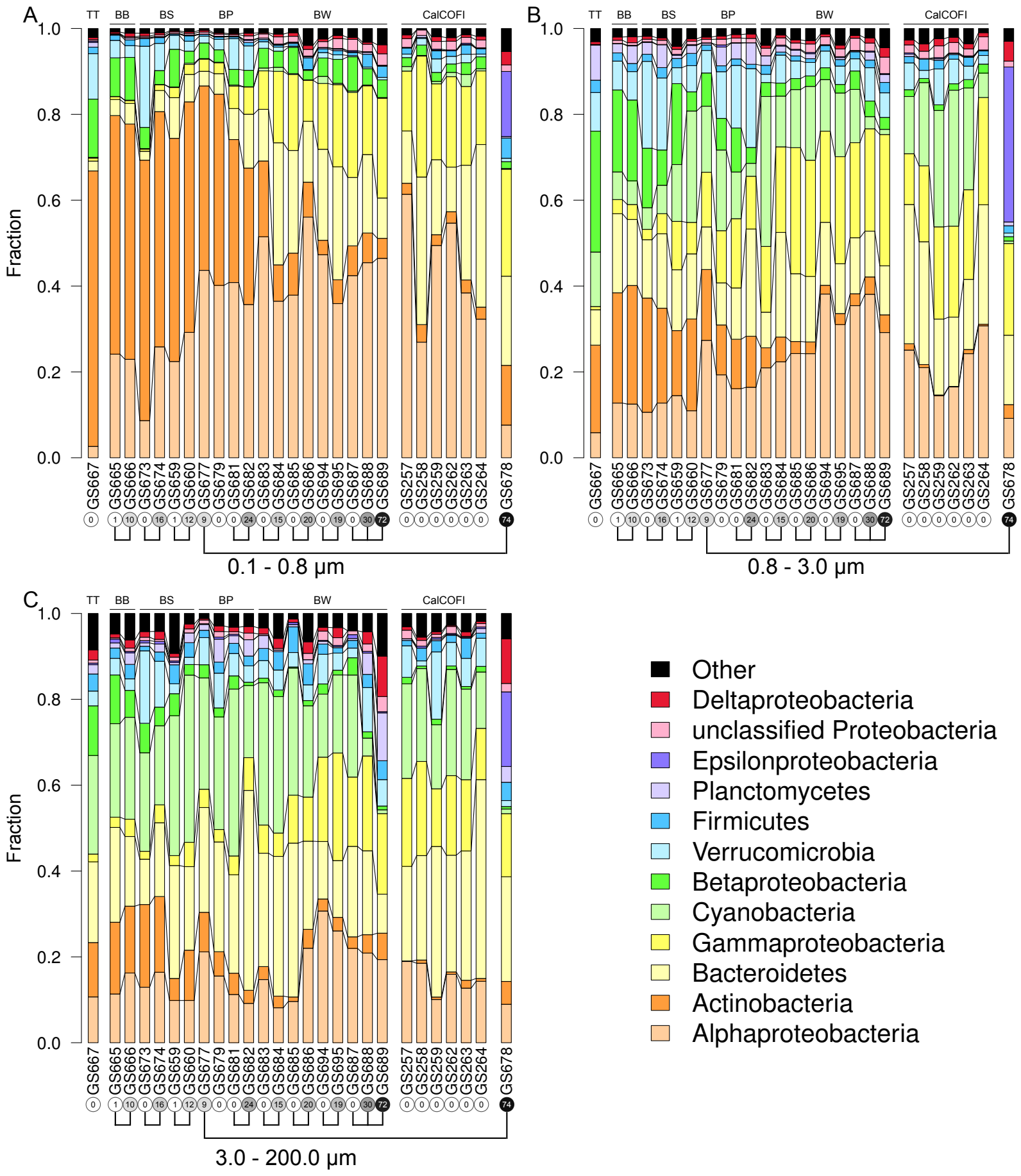

Supplement: Figure S4 — Taxonomic composition of bacteria in the A) 0.1–0.8 µm, B) 0.8–3.0 µm, and C) 3.0–200 µm size fractions at each site. The same bacterial phyla/classes as in Figure 2A are shown. Sampling depth (m) is indicated within circles below sample names, with interconnecting circles indicating samples from the same location. The corresponding basin for samples is shown in the top margin: TT = Torne Träsk, BB = Bothnian Bay, BS = Bothnian Sea, BP = Baltic Proper, BW = Baltic West. CalCOFI represents samples taken from the coastal eastern Pacific. (PDF) [file pone.0089549.s004.pdf]

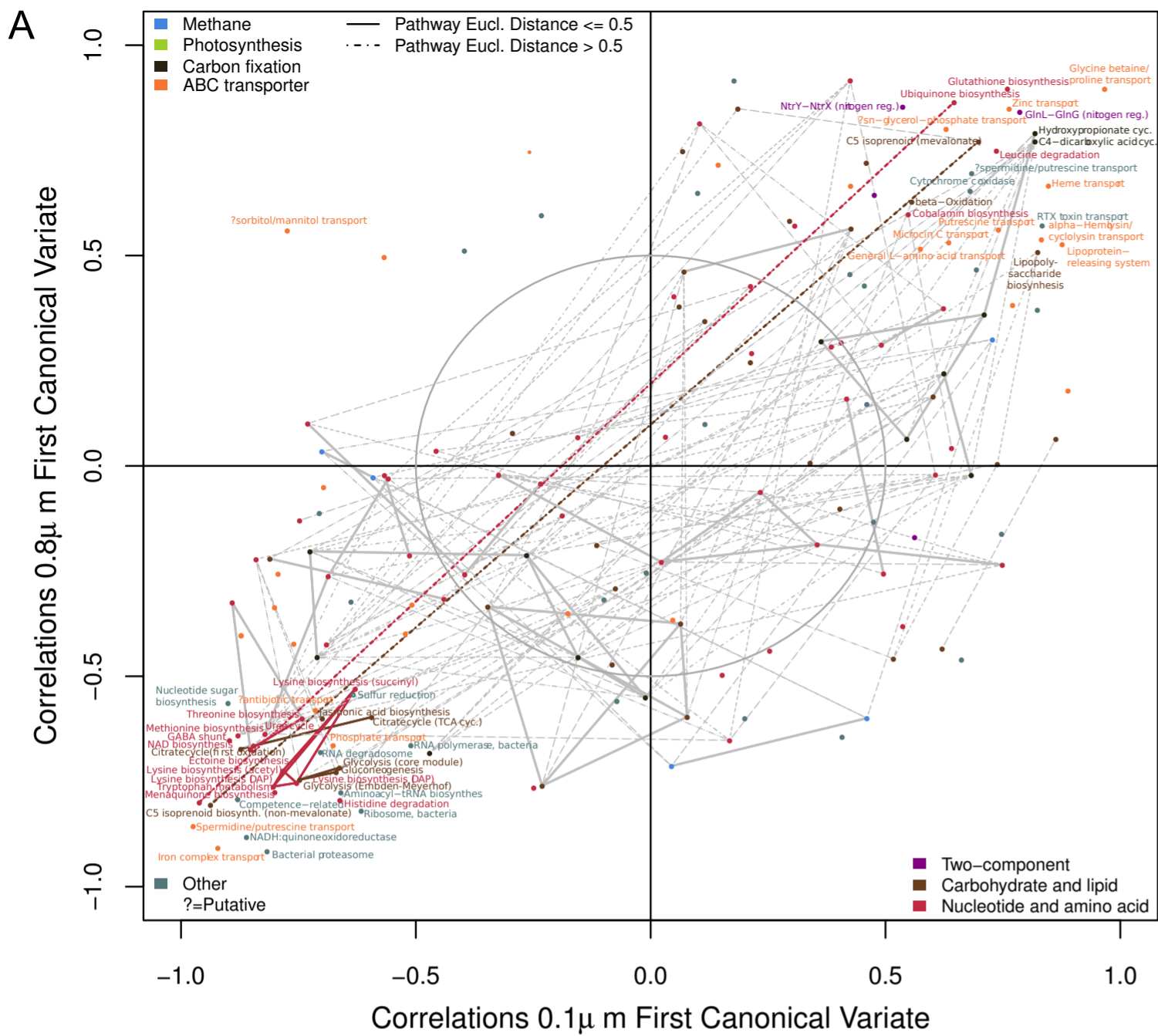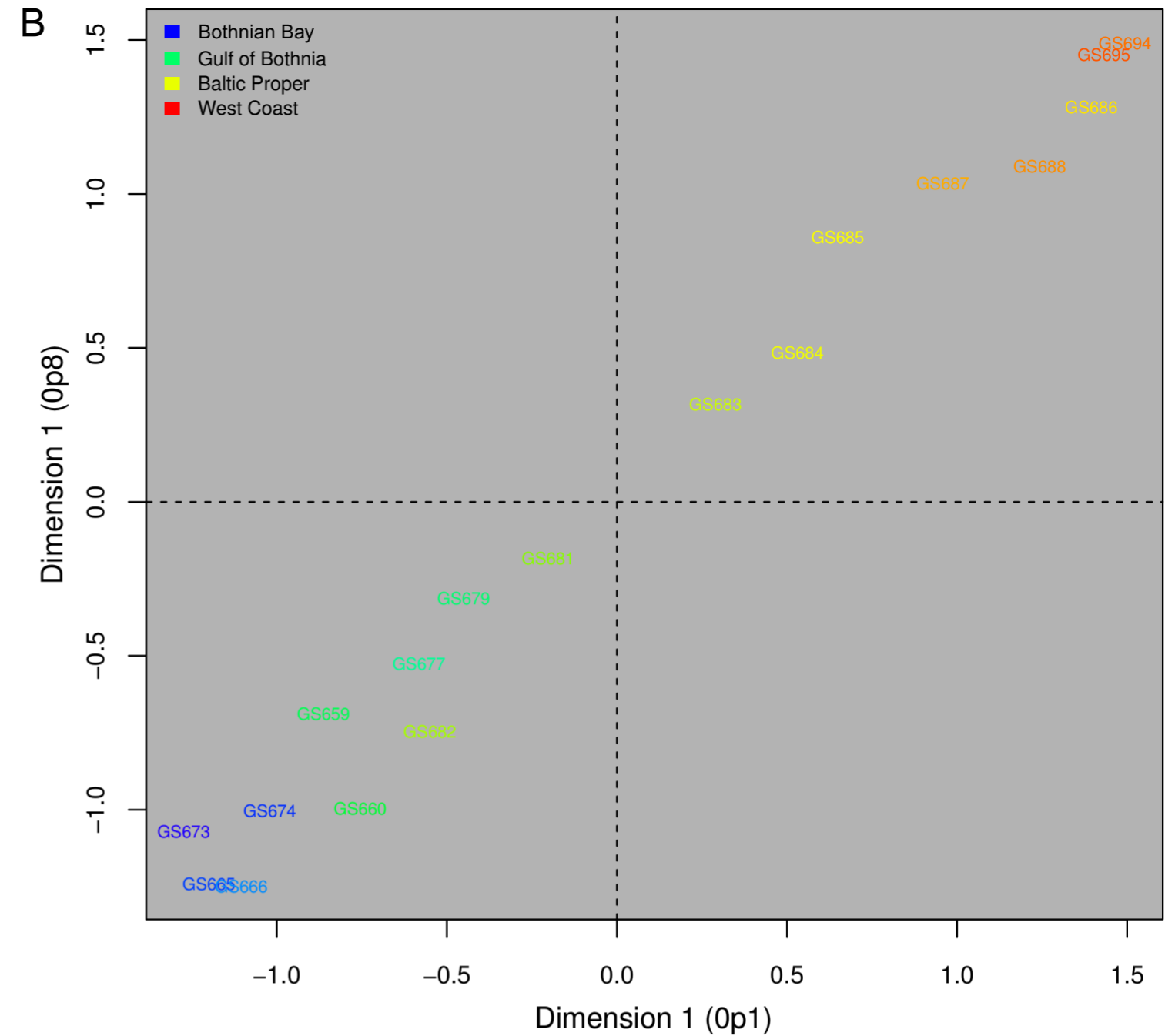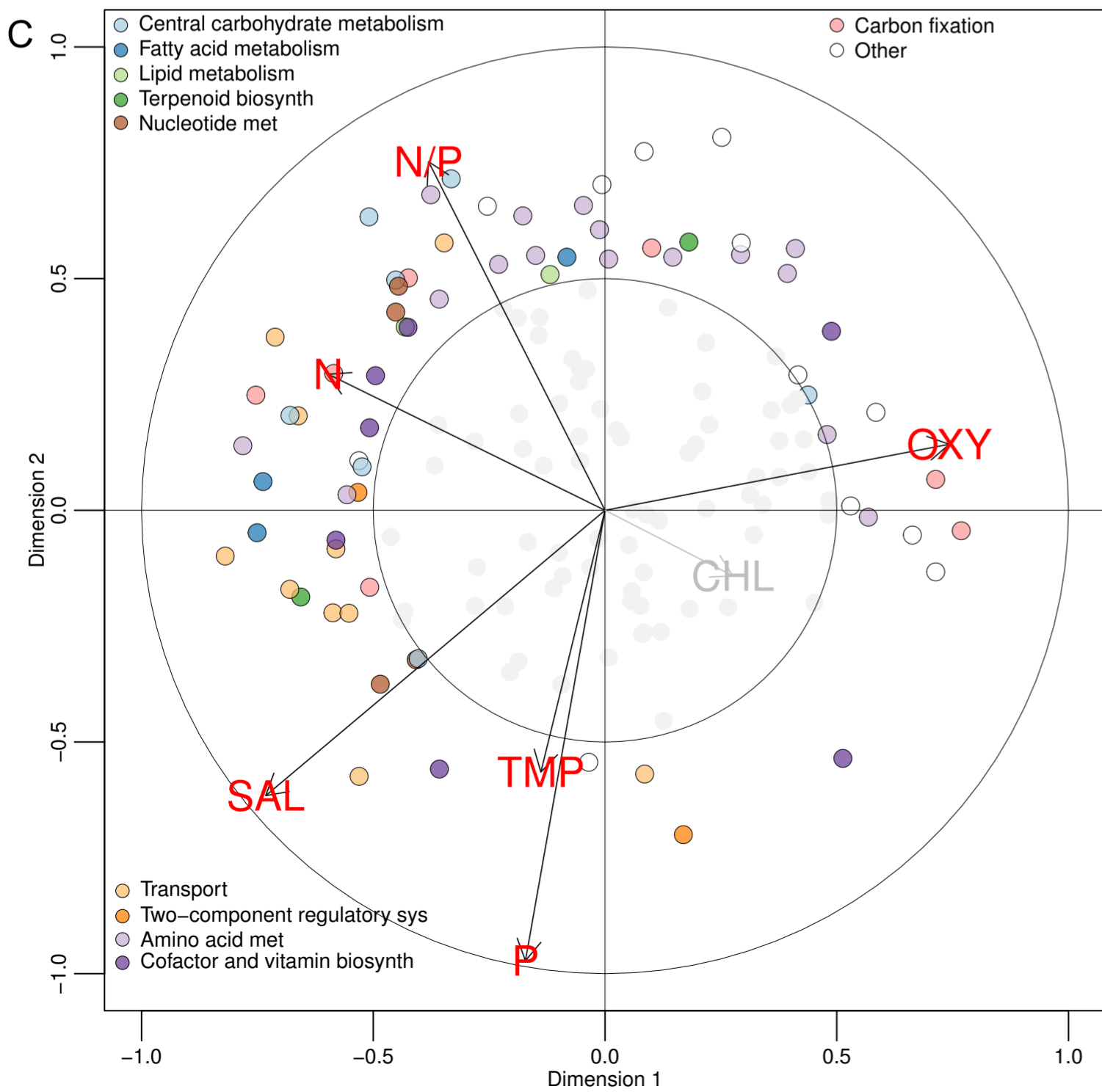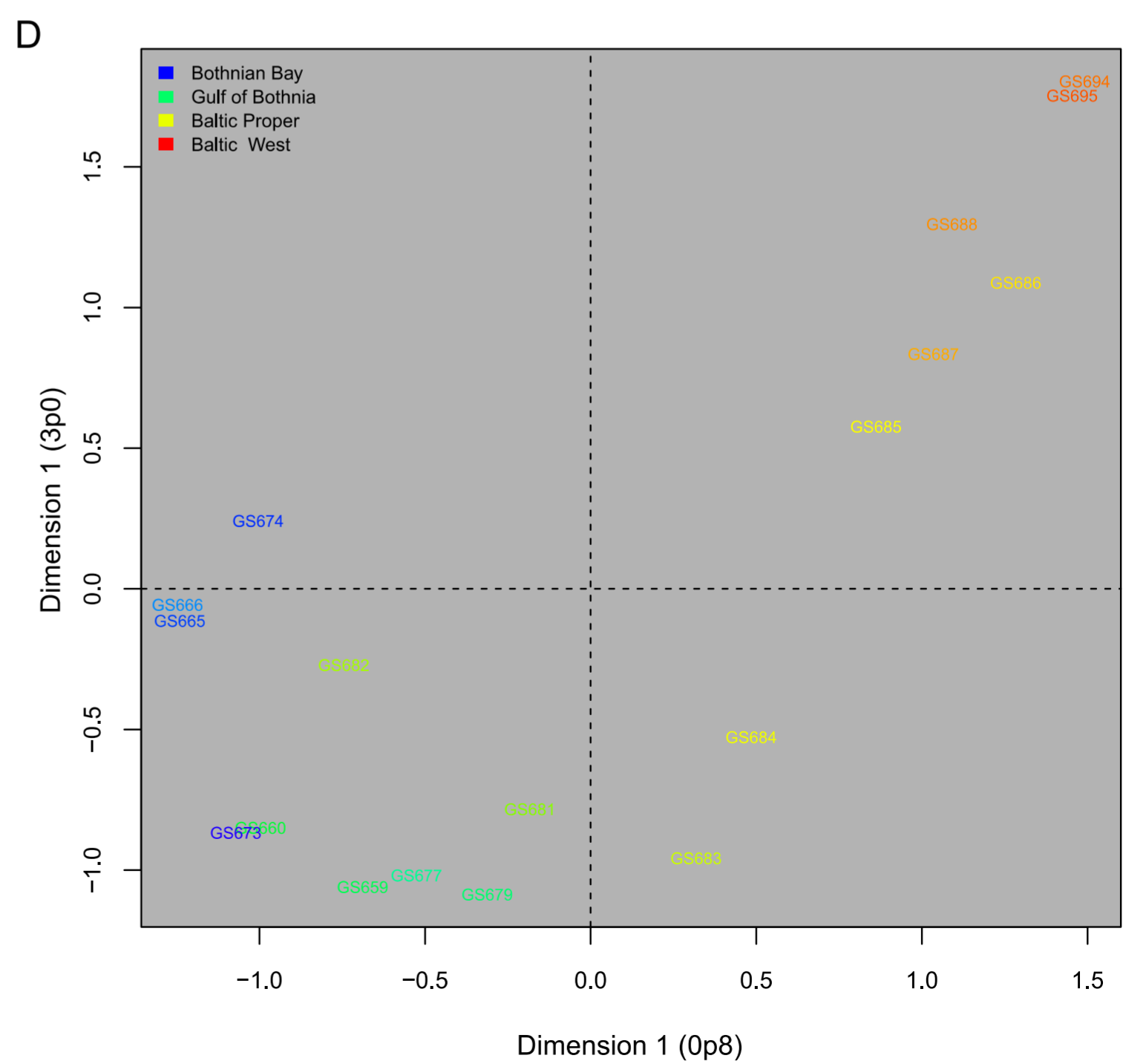

Supplement: Figure S6 — A) Correlation of functional modules against first variant M1 for both 0.1 and 0.8 um communities. B) A sample by sample comparison of the 1st dimension of 0.1 (x axis) and 0.8 (y axis) fractions. C) Module and metadata correlations with first and second canonical variates for the bacterial communities found in the 3.0–200 µm fraction. D) A sample by sample comparison of the 1st dimension of 0.8 and 3.0 fractions. (PDF) [file pone.0089549.s006.pdf]

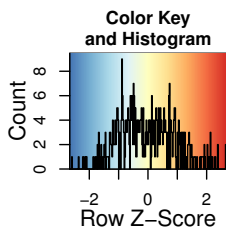

TT BB BS BP BW BP

GS667 GS665 GS666 GS673 GS674 GS659 GS660 GS677 GS679 GS681 GS682 GS683 GS684 GS685 GS686 GS694 GS695 GS687 GS688 GS689 GS678

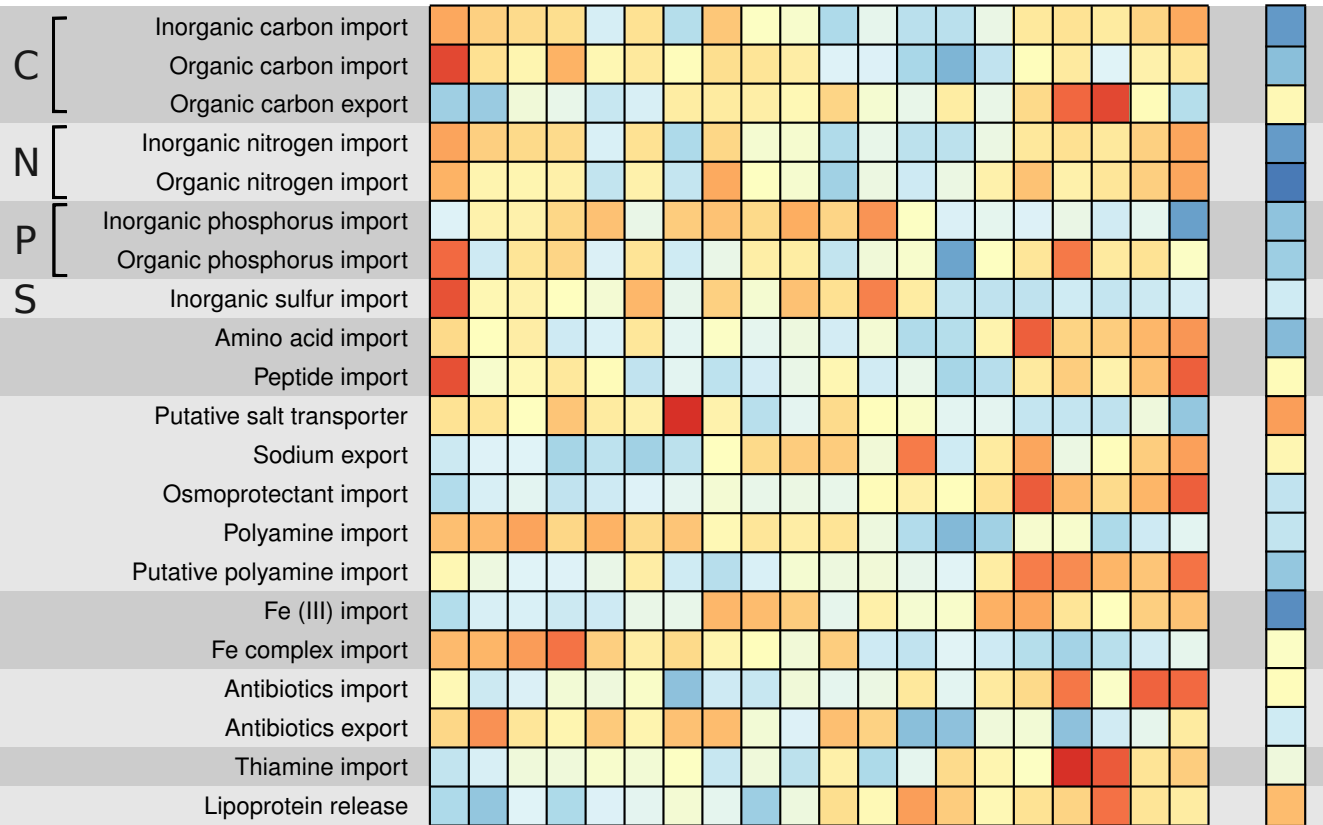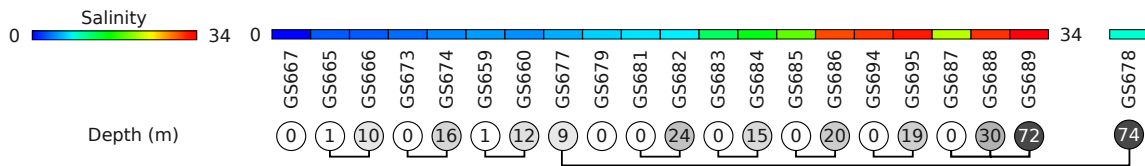

Supplement: Figure S8 — Distribution of prokaryotic-type ABC transporters in the Baltic dataset (see Methods for details). Heatmap color scaling is based on rows. *“Putative salt transporter” has been suggested to be involved in salt transport and/or cell division. (PDF) [file pone.0089549.s008.pdf]

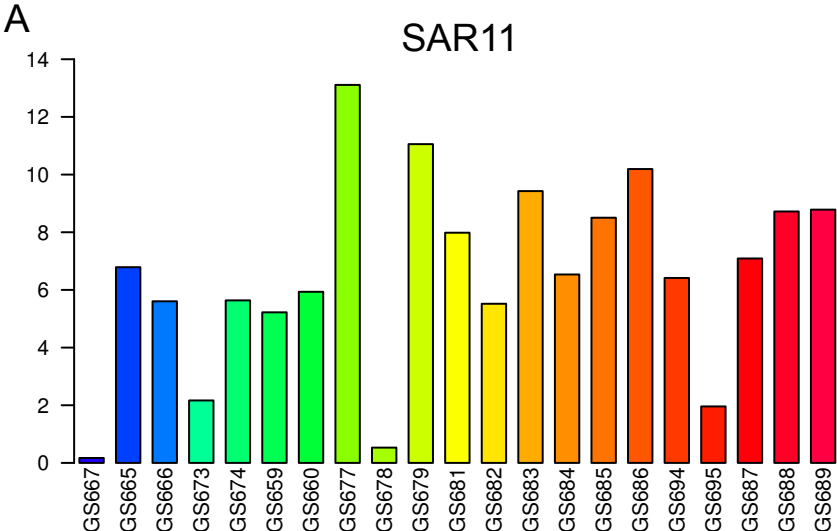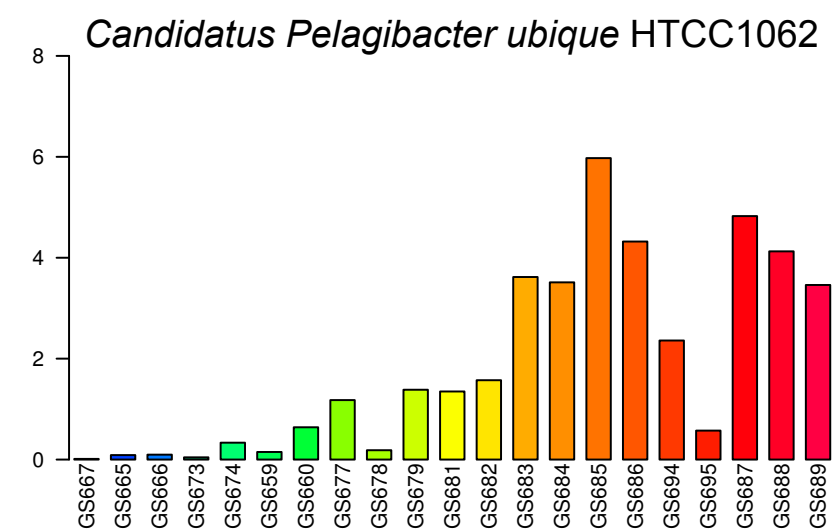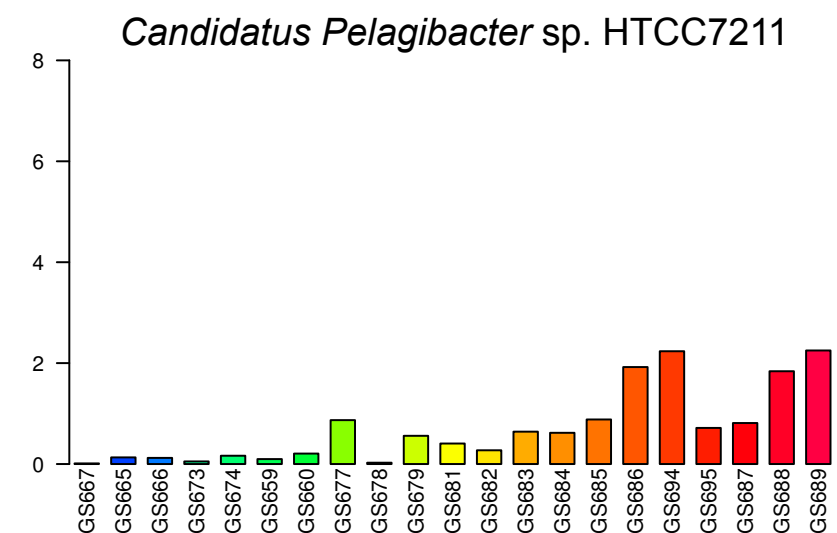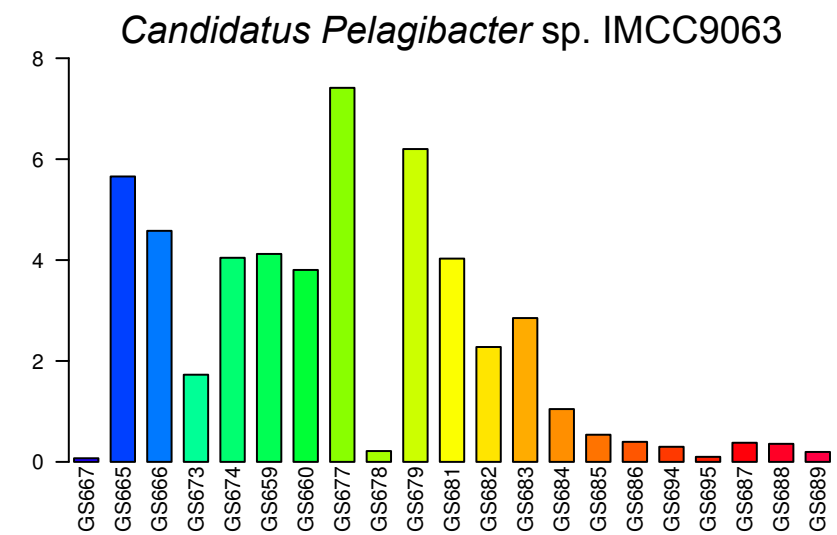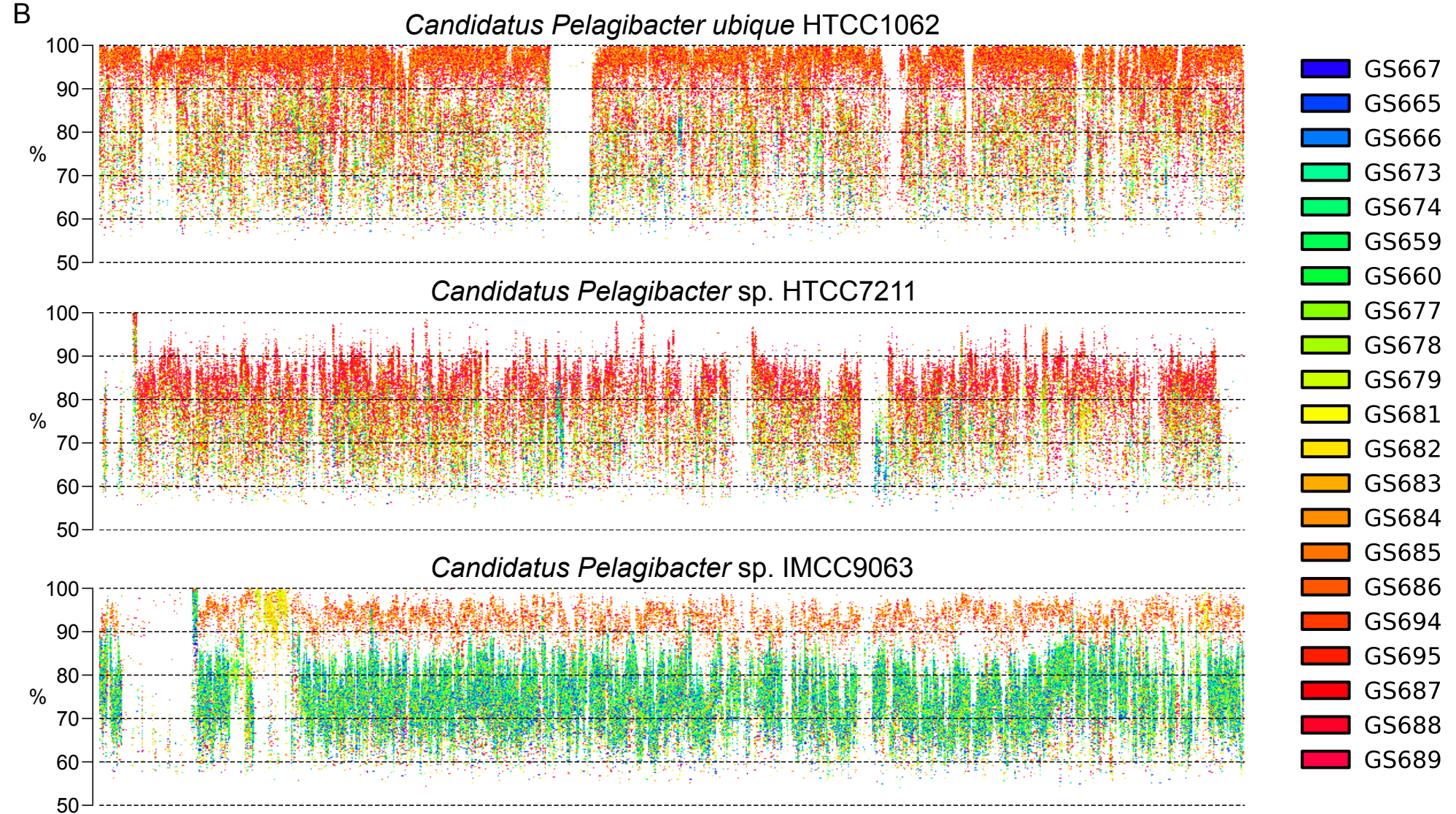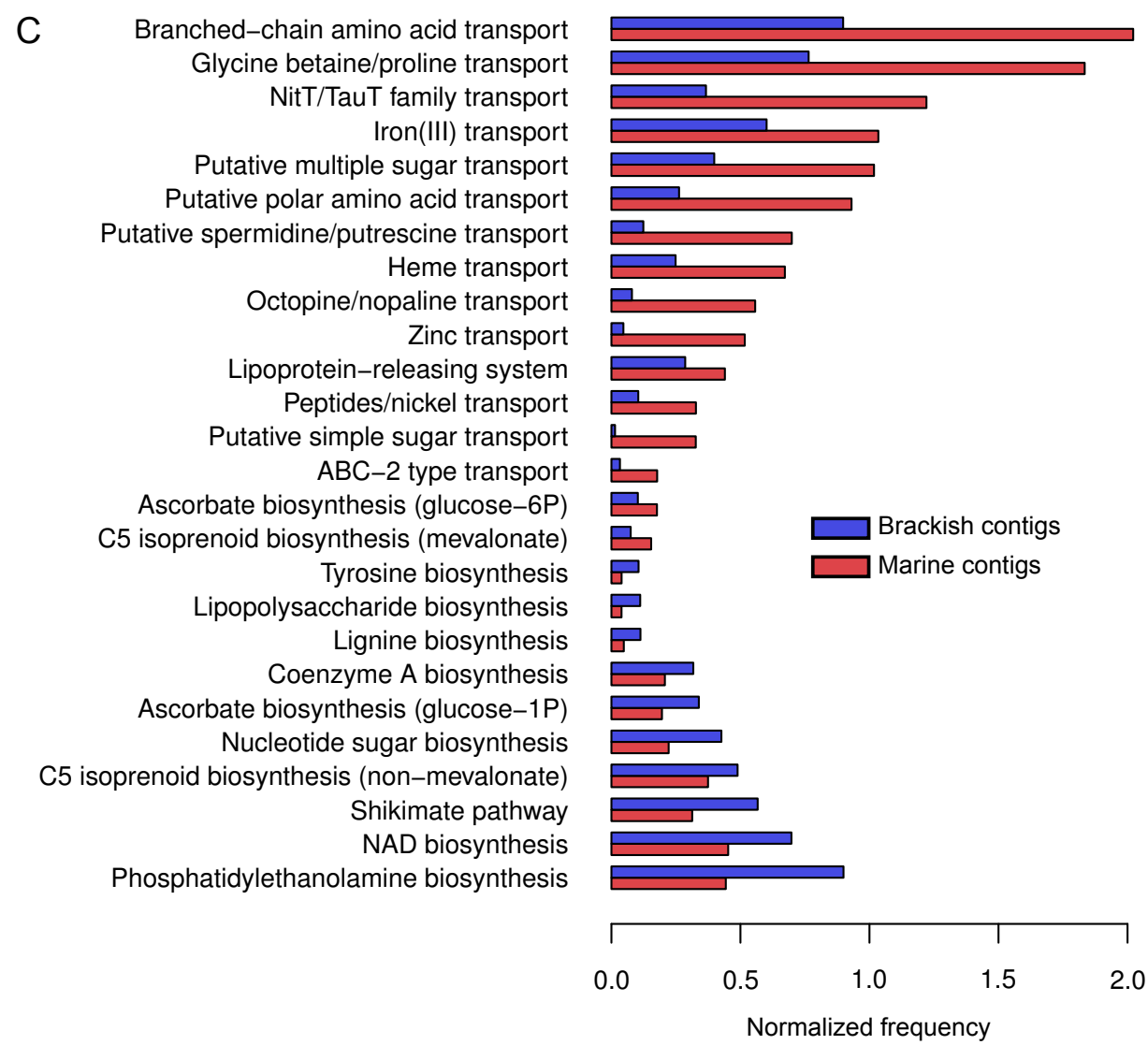

Supplement: Figure S10 — A) Annotations of SAR11 by APIS. SAR11 (Pelagibacterales) are found across the entire transect, but in a species and clade specific manner. Clade IIIa (IMCC9063) is primarily found in the low salinity environments while clade Ia (Can. Pelagibacter ubique and HTCC7211) dominates the high salinity environment. B) Fragment recruitment plots of different Pelagibacterales genomes. While IMCC9063 is the most similar to those found in the low salinity waters (cold colors), the population in this region is only 70–80% average nucleotide identity and should be considered a distinct species and possibly sub-clade. C) Frequency of functional modules related to transport and biosynthesis in the SAR11-like combined assembly. Contigs were divided into brackish and marine bins based on read proportion from samples in the different environments (brackish: GS665-GS682, marine: GS683-GS695). (PDF) [file pone.0089549.s010.pdf]
