# Supplementary material for: Functional Tradeoffs Underpin Salinity-Driven Divergence in Microbial Community Composition
Source: PLoS One. 2014 Feb 27;9(2):e89549. doi: 10.1371/journal.pone.0089549 (PMC3937345; doi:10.1371/journal.pone.0089549)
Supplement: Figure S5 — Regularized canonical correlation analysis of the abundance of bacterial phylotypes at the genus level. For ease of visualization, each genus is color coded according to Phylum. The analysis recapitulates many of the trends from the Phyla level analysis, with actinobacteria, verrucamicrobia, and many bacteroidetes being found at low salinity, while gamma and alpha proteobacterial are found at high. This analysis does provide more detailed information, such as the alphaproteobacteria in the SAR11 lineage, HIMB114, being found at low salinity that is recapitulated by other analyses (Fig. 5A). (PDF) [file pone.0089549.s005.pdf]

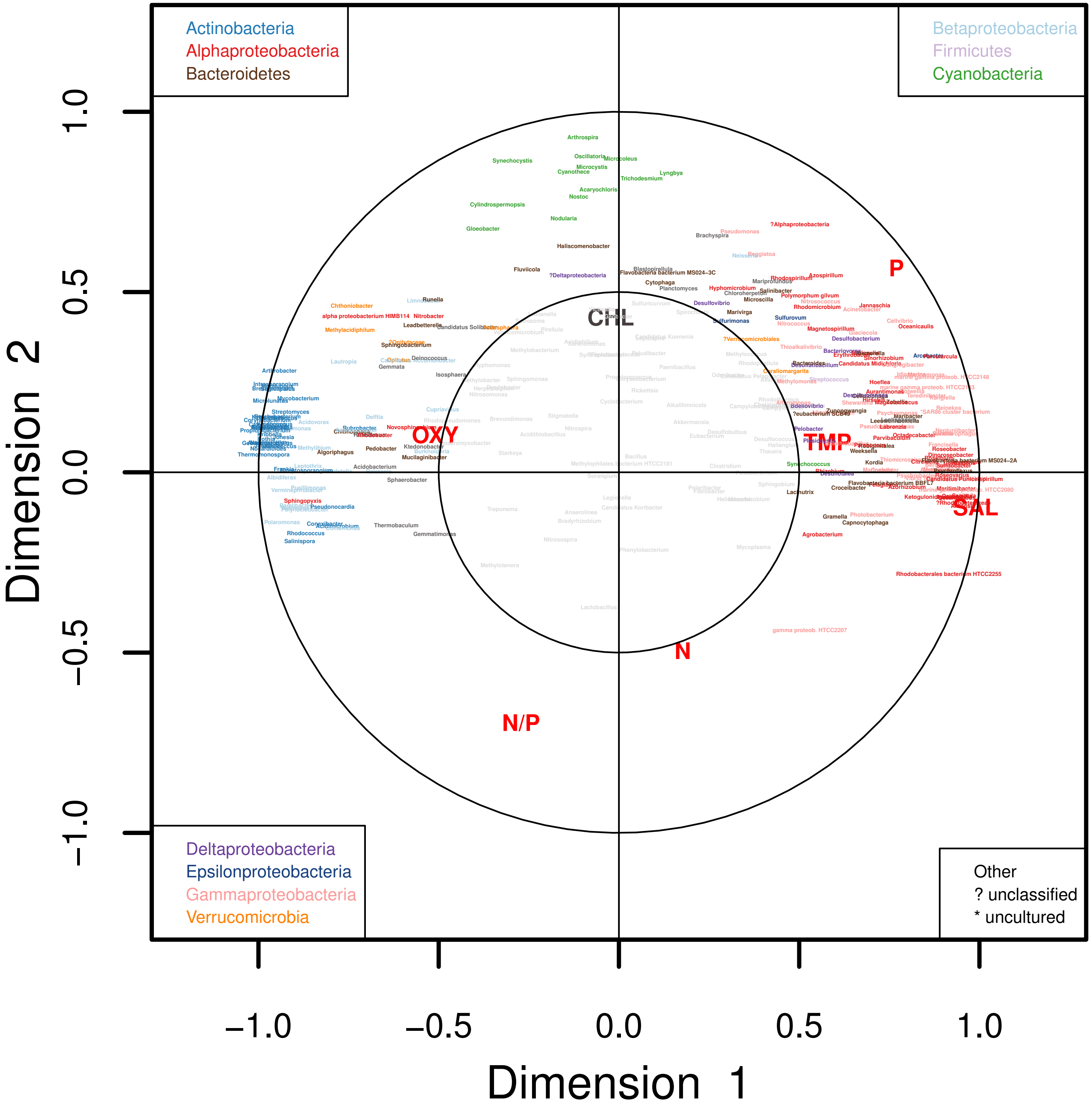
Actinobacteria
Alphaproteobacteria
Bacteroidetes
Betaproteobacteria
Firmicutes
Cyanobacteria
Deltaproteobacteria
Epsilonproteobacteria
Gammaproteobacteria
Verrucomicrobia
Other
? unclassified
* uncultured
P
CHL
OXY
TMP
SAL
N
N/P
Dimension 2
Dimension 1
1.0
0.5
0.0
−0.5
−1.0
−1.0
−0.5
0.0
0.5
1.0
