# Supplementary material for: Functional Tradeoffs Underpin Salinity-Driven Divergence in Microbial Community Composition
Source: PLoS One. 2014 Feb 27;9(2):e89549. doi: 10.1371/journal.pone.0089549 (PMC3937345; doi:10.1371/journal.pone.0089549)
Supplement: Figure S7 — A schematic of community scale metabolic adaptations across salinity gradients. Note that while the metabolic pathways are not expressly linked, they share substrates and products and thus have the potential. (PDF) [file pone.0089549.s007.pdf]

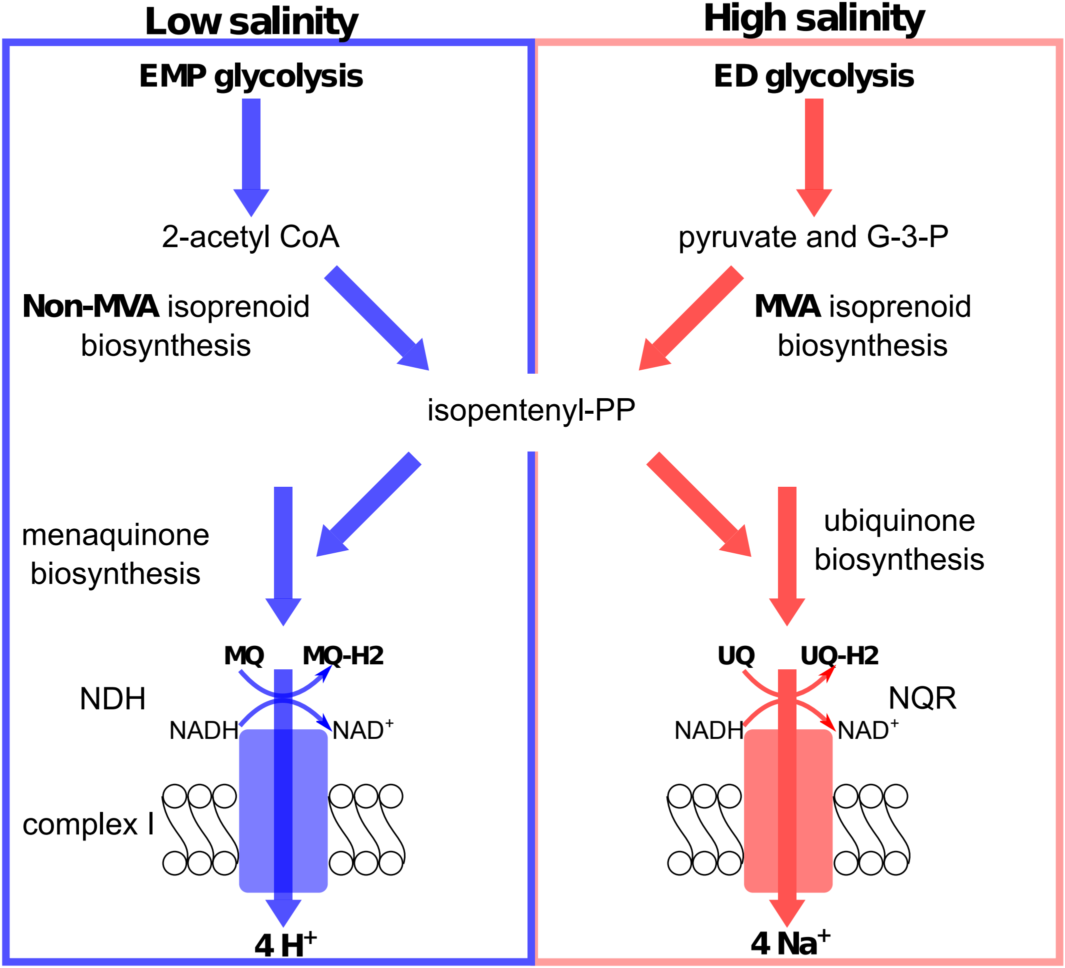

Low salinity
High salinity
EMP glycolysis
ED glycolysis
2-acetyl CoA
pyruvate and G-3-P
Non-MVA isoprenoid biosynthesis
MVA isoprenoid biosynthesis
isopentenyl-PP
menaquinone biosynthesis
ubiquinone biosynthesis
MQ
MQ-H2
UQ
UQ-H2
NDH
NQR
NADH
NAD+
NADH
NAD+
complex I
4 H+
4 Na+
