# Supplementary material for: Functional Tradeoffs Underpin Salinity-Driven Divergence in Microbial Community Composition
Source: PLoS One. 2014 Feb 27;9(2):e89549. doi: 10.1371/journal.pone.0089549 (PMC3937345; doi:10.1371/journal.pone.0089549)
Supplement: Figure S9 — Regularized canonical correlation analysis of functional modules within the 9 most abundant bacterial phyla or classes in the total dataset. For each plot, the outer circle (1) and inner circles (0.5) display the amount of variance explained by the linear combinations of variables. The variance explained for points within the inner circle is less than 25%, thus these have been dimmed. Labelled arrows show the correlation coefficient of environmental variables. Each point represents a KEGG functional module and is colored according to its parent metabolic category (see KEGG Metabolic Category legend). Specific modules discussed in the text are indicated in each plot by numbers (1–13) and described in the KEGG Module legend with colored circles matching their parent metabolic category. (PDF) [file pone.0089549.s009.pdf]

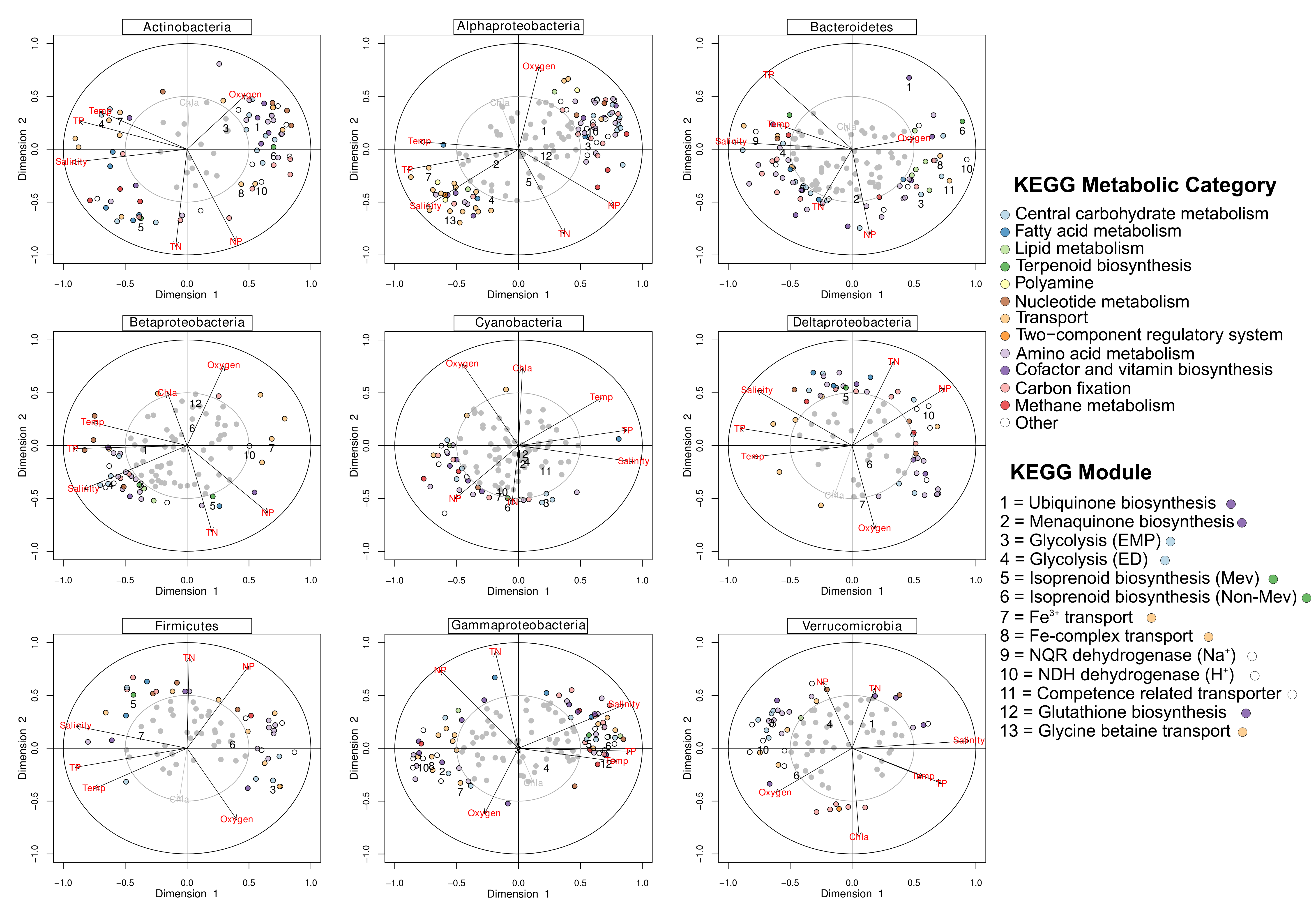

Actinobacteria
Alphaproteobacteria
Bacteroidetes
Betaproteobacteria
Cyanobacteria
Deltaproteobacteria
Firmicutes
Gammaproteobacteria
Verrucomicrobia
Dimension 1
Dimension 2
Oxygen
Temp
TP
TN
NP
Salinity
Chla
KEGG Metabolic Category
Central carbohydrate metabolism
Fatty acid metabolism
Lipid metabolism
Terpenoid biosynthesis
Polyamine
Nucleotide metabolism
Transport
Two−component regulatory system
Amino acid metabolism
Cofactor and vitamin biosynthesis
Carbon fixation
Methane metabolism
Other
KEGG Module
1 = Ubiquinone biosynthesis
2 = Menaquinone biosynthesis
3 = Glycolysis (EMP)
4 = Glycolysis (ED)
5 = Isoprenoid biosynthesis (Mev)
6 = Isoprenoid biosynthesis (Non-Mev)
7 = Fe^{3+} transport
8 = Fe-complex transport
9 = NQR dehydrogenase (Na^{+})
10 = NDH dehydrogenase (H^{+})
11 = Competence related transporter
12 = Glutathione biosynthesis
13 = Glycine betaine transport
