# Supplementary material for: Functional Tradeoffs Underpin Salinity-Driven Divergence in Microbial Community Composition
Source: PLoS One. 2014 Feb 27;9(2):e89549. doi: 10.1371/journal.pone.0089549 (PMC3937345; doi:10.1371/journal.pone.0089549)
Supplement: Table S1 — Sites and environmental data for the sites where metagenomic sequencing was conducted. (DOC) [file pone.0089549.s011.doc]

**Table S1**: Sites and environmental data for the sites where metagenomic sequencing was conducted.

| Station | Description | Lat | Lon | Depth (m) | Temp  (°C) | Salinity (PSU) | Oxygen (%sat.) | Total N (mM) | Total P (mM) | N:P | Chl a (mg/L) |
| --- | --- | --- | --- | --- | --- | --- | --- | --- | --- | --- | --- |
| GS659 | Bottenhavet, Station C-3 | 62.65417 | 18.9475 | 1 | 15.31 | 4.79 | 110 | 0.885 | 0.201 | 4.412 | 0.6 |
| GS660 | Bottenhavet, Station C-3 | 62.65417 | 18.9475 | 12 | 8.02 | 5.09 | 102 | 1.184 | 0.187 | 6.344 | 1.48 |
| GS665 | Bottenviken, Station A5 | 65.16733 | 23.23233 | 1 | 10.80 | 2.9 | 89 | 3.033 | 0.188 | 16.156 | 1.5 |
| GS666 | Bottenviken, Station A5 | 65.16817 | 23.23233 | 10 | 9.10 | 2.9 | 94 | 3.697 | 0.153 | 24.240 | 1.97 |
| GS667 | Lake Torne Träsk, Abisko, Sweden | 68.35584 | 18.82167 | 1 | 7.89 | 0 | 95 | 2.909 | 0.162 | 17.987 | 1 |
| GS673 | N. Kvarken, Station B-3 | 63.49542 | 19.8135 | 1 | 14.90 | 3.46 | 95 | 0.788 | 0.208 | 3.793 | 1.33 |
| GS674 | N. Kvarken, Station B-3 | 63.49542 | 19.8135 | 16 | 10.30 | 4.51 | 80 | 1.181 | 0.189 | 6.255 | 0.92 |
| GS677 | Helcom site 4, Deepest section of the Baltic Sea | 58.58123 | 18.2328 | 9 | 15.31 | 5.75 | 98.5 | 0.871 | 0.283 | 3.079 | 5 |
| GS678 | Helcom site 4, Deepest section of the Baltic Sea | 58.58123 | 18.2328 | 74 | 5.32 | 9.9 | 1 | 2.677 | 2.267 | 1.181 | 0.42 |
| GS679 | Off south tip of Öland, bloom occuring | 56.1664 | 16.37822 | 1 | 19.39 | 6.89 | 98 | 1.151 | 0.421 | 2.734 | 0.97 |
| GS681 | German Baltic Sample, IOW Station | 54.9295 | 13.48222 | 1 | 17.56 | 7.52 | 97 | 1.975 | 0.424 | 4.658 | 1.56 |
| GS682 | German Baltic Sample, IOW Station | 54.9295 | 13.48222 | 24 | 9.47 | 8 | 76.5 | 1.003 | 0.395 | 2.539 | 0.54 |
| GS683 | Danish sample site DMU 939 | 54.57023 | 11.33218 | 1 | 18.15 | 13.72 | 99.5 | 0.757 | 0.458 | 1.654 | 2.41 |
| GS684 | Danish sample site DMU 939 | 54.57023 | 11.33218 | 15 | 17.00 | 16.45 | 91.5 | 0.904 | 0.495 | 1.826 | 4.52 |
| GS685 | Kattegat-Helcom site | 56.66342 | 12.11832 | 1 | 18.63 | 20.22 | 92.2 | 0.704 | 0.258 | 2.728 | 0.87 |
| GS686 | Kattegat-Helcom site | 56.66342 | 12.11832 | 20 | 15.00 | 31-5 | 88 | 2.205 | 0.360 | 6.121 | 1.64 |
| GS687 | Lyskil Fjord-Alsbäck station | 58.31767 | 11.54037 | 1 | 18.85 | 23.2 | 83 | 1.294 | 0.382 | 3.391 | 2.95 |
| GS688 | Lyskil Fjord-Alsbäck station | 58.31767 | 11.54037 | 30 | 13.54 | 32.4 | 77.7 | 2.321 | 0.461 | 5.034 | 0.43 |
| GS689 | Lyskil Fjord-Alsbäck station | 58.31767 | 11.54037 | 72 | 6.35 | 34.35 | 60 | 9.608 | 1.172 | 8.196 | 0.32 |
| GS694 | DMU 1005, Denmark | 57.79782 | 10.87143 | 1 | 17.43 | 32.16 | 84 | 3.810 | 0.335 | 11.389 | 0.8 |
| GS695 | DMU 1005, Denmark | 57.79782 | 10.87143 | 19 | 17.47 | 33.05 | 80 | 2.439 | 0.345 | 7.070 | 1.34 |
