# Supplementary material for: Functional Tradeoffs Underpin Salinity-Driven Divergence in Microbial Community Composition
Source: PLoS One. 2014 Feb 27;9(2):e89549. doi: 10.1371/journal.pone.0089549 (PMC3937345; doi:10.1371/journal.pone.0089549)
Supplement: Table S2 — Number of peptides generated from pyrosequencing reads for each filter at each site, along with the portion that could be annotated using the JCVI metagenomic annotation pipeline. (DOC) [file pone.0089549.s012.doc]

**Table S2**: Number of peptides generated from pyrosequencing reads for each filter at each site, along with the portion that could be annotated using the JCVI metagenomic annotation pipeline.

| Station | 0.1 peptides | %ann | 0.8 peptides | %ann | 3.0 peptides | %ann | Viral peptides | %ann |
| --- | --- | --- | --- | --- | --- | --- | --- | --- |
| GS659 | 299,806 | 53% | 622,158 | 33% | 524,577 | 14% |  |  |
| GS660 | 514,707 | 48% | 463,444 | 34% | 267,124 | 16% |  |  |
| GS665 | 349,492 | 54% | 247,307 | 39% | 227,441 | 12% |  |  |
| GS666 | 220,067 | 54% | 434,004 | 47% | 403,074 | 17% |  |  |
| GS667 | 214,501 | 60% | 176,227 | 46% | 203,219 | 20% | 90,600 | 42% |
| GS673 | 406,191 | 44% | 643,058 | 44% | 140,063 | 19% |  |  |
| GS674 | 196,512 | 53% | 216,044 | 43% | 212,166 | 25% |  |  |
| GS677 | 277,800 | 51% | 367,670 | 39% | 352,574 | 16% |  |  |
| GS678 | 300,548 | 56% | 527,400 | 51% | 472,424 | 34% | 358,515 | 47% |
| GS679 | 338,924 | 57% | 1,077,371 | 38% | 226,197 | 27% | 319,667 | 70% |
| GS681 | 244,188 | 48% | 399,163 | 40% | 355,476 | 22% |  |  |
| GS682 | 364,486 | 40% | 365,908 | 47% | 151,250 | 12% |  |  |
| GS683 | 343,192 | 42% | 330,750 | 25% | 463,651 | 14% |  |  |
| GS684 | 208,587 | 45% | 483,249 | 41% | 233,578 | 12% |  |  |
| GS685 | 330,549 | 51% | 755,990 | 35% | 218,732 | 12% |  |  |
| GS686 | 147,524 | 50% | 273,029 | 34% | 426,463 | 13% |  |  |
| GS687 | 292,645 | 45% | 266,710 | 38% | 184,563 | 12% |  |  |
| GS688 | 269,074 | 51% | 251,408 | 44% | 409,554 | 23% |  |  |
| GS689 | 271,888 | 53% | 268,904 | 47% | 174,710 | 27% | 175,393 | 45% |
| GS694 | 205,836 | 22% | 214,378 | 40% | 205,818 | 22% |  |  |
| GS695 | 347,179 | 48% | 355,802 | 30% | 310,216 | 24% | 398,293 | 79% |
