# Supplementary material for: Functional Tradeoffs Underpin Salinity-Driven Divergence in Microbial Community Composition
Source: PLoS One. 2014 Feb 27;9(2):e89549. doi: 10.1371/journal.pone.0089549 (PMC3937345; doi:10.1371/journal.pone.0089549)
Supplement: Materials and Methods S1 — Detailed description of materials and methods. (DOC) [file pone.0089549.s014.doc]

# Supplementary Materials and Methods

## Sample collection, sequencing and annotation

Samples were collected during the European leg of The Sorcerer II Global Ocean Sampling expedition in July 2009 with parallel environmental characterization using an integrated CTD-oxygen, chlorophyll a, and pH sensor package. Between 100-200 L of water was pre-filtered with 200 µm mesh and then fractionated into three different size classes; 0.1-0.8 µm, 0.8-3.0 µm and 3.0-200 µm. Filters were stored at -80°C in protective buffer (10 mL sterile filtered sampling water, 10 mL RNAlater, 200 µl 100x TE buffer, 400 µl 0.5M EGTA and 400 µl 0.5M EDTA) until DNA extraction [1]. Filtered water was retained and circulated in a Tangential Flow Filtration (TFF) system with a 50 kDa cut-off for concentration of viruses. Samples for nutrient analyses were collected in acid washed HDPE bottles after 200 µm prefiltration and frozen at -20°C prior to analyses by the Virginia Institute of Marine Science analytical center.

Each size fraction was shotgun-sequenced using pyrosequencing. The dataset comprises 7.16 billion nucleotides in 20.9 million reads (avg. length = 324 bp), with 22,914,138 translated amino acid sequences (Table S2). A merged set of seven microbial metagenomes with similar size fractionation-based sequencing from the west coast of California (USA) [1] was used as an oceanic outgroup for many analyses. Reads were annotated using the JCVI metagenomic annotation pipeline [2], along with APIS [1] and fragment recruitment [3]. A global assembly of all sequence data was performed using the Newbler Assembler [4], with a final assembly of 490 million basepairs, a N50 of 1.5 Kbp, and a largest contig of 106 Kbp. Approximately 15,000 16S rRNA subunit amplicons were also sequenced for each location and size fraction and phylogenetically annotated as described previously [5]. All sequence and annotation data is available at CAMERA [6] under accession number CAM_P_0001109 and Metarep [7] (metarep.jcvi.org). Core marker proteins were placed into maximum likelihood reference trees, and genome size estimates were calculated as described previously [1].

## Determination of bacterial community composition

The AMPHORA2 software package [8] was used to identify and align metagenomic protein sequences in the Baltic and reference datasets. Default trimming was applied during alignment to the Amphora reference HMMs and metagenomic sequences consisting of all-gap columns after trimming were removed. Sequences were placed individually onto the corresponding Amphora reference phylogenies using the maximum likelihood (ML) method in RAxML [9,10] (v. 7.3.0) with the CAT approximation, the WAG amino acid substitution model [11] and with an estimated proportion of invariable sites (PROTCATIWAG) with subsequent classification of reads using the Phylotyping.pl script in Amphora. The frequency of bacterial phyla were calculated as averages from phylotyping results of all 31 Amphora marker genes. Sequences with likelihood weights <0.7 at domain Bacteria were excluded from the frequency calculation (6,381 of 152,838 total sequences excluded). Correlations between Amphora, APIS and 16S rRNA gene amplicon results were analysed using the Pearson's product moment correlation coefficient.

## Regularized Canonical Correlation Analysis (RCCA)

Environmental variables were obtained from processed CTD or nutrient data (table S1). For the phylum and genus level analyses, pooled sample proportions were obtained for each phylum variable by adding up APIS classification counts across the three filter fractions (for eukaryotic phyla only the 0.8 µm and 3.0 µm filters were used due to noise added by the 0.1 µm filter data) followed by normalization by the total pooled domain APIS counts (classified as eukaryotes, bacteria, or viral respectively). For the module analysis sample proportions were obtained for a subset of modules (eukaryotic and archaea specific modules were excluded) by taking the average KEGG Ortholog (KO) frequencies, normalized as described previously [12], of all KOs in a module. An aggregated proportion across the three filter fractions was obtained by summing-up the average module proportions. To make the phyla/module variable distributions more symmetric, proportions were log transformed. To account for differences in the normalized count range and to obtain coefficients of determination, variables were standardized (mean=0, std=1). For the same reasons, metadata variables (Salinity, Temperature, Total Phosphorus, Total Nitrogen, N:P ratio, Oxygen, Temperature) were log transformed and standardized (no normalization was applied). Variables for which univariate normality was rejected (Shapiro-Wilk p-value < 0.01) were discarded.

### Outlier Detection

Three out of the 21 samples with extreme metadata profiles based on boxplots and bivariate normality plots were excluded (GS678, GS667, GS689). No extreme outliers (≥ 3 standard deviations) were observed for phyla/module variables for the remaining 18 samples.

### Variable Selection

For the phylum analysis, the top 20 bacterial phyla based on normalized counts across the 18 samples were selected. 7 eukaryotic and 5 viral phyla were chosen based on informed selection. Modules with an overall total (across all 18 datasets) of >0.05% of the sum of all normalized module counts were retained. Resulting variable sets were used for input to the RCCA analysis (r-package CCA). Regularization parameters were estimated using one-fold cross validation.

### NQR and NDH dehydrogenases

The frequencies of the Na+ translocating (NQR) and the type I and II NADH dehydrogenases were calculated from Kegg ortholog (KO) frequencies, normalized as above. The NQR type included KOs K00346-51 while the type I and type II dehydrogenases included KOs K00330-43, K13378, K13380, K15863 and K03885, respectively. Average KO frequencies were calculated for each type with combined averages for the type I and II frequencies.

## SAR11 analysis

### Phylogenetic analysis

Protein sequences for all bacterial marker genes in the Amphora package were obtained from eight SAR11 genomes: Ca. Pelagibacter HTCC1062 and HTCC1002 [13], HTCC7211, IMCC9063 [14], and alphaproteobacterium HIMB114, HIMB59 and HIMB5 [15]. Seven Rickettsiales genomes were selected as an outgroup, based on the common SAR11 sistergroup in the Amphora reference phylogenies: Ehrlichia ruminantium str. Welgevonden, Ehrlichia ruminantium str. Gardel, Ehrlichia canis str. Jake, Anaplasma phagocytophilum HZ, Neorickettsia sennetsu str. Miyayama, Ehrlichia chaffeensis str. Arkansas and Neorickettsia risticii str. Illinois. Protein sequences for the 31 Amphora marker genes were aligned to the respective reference HMM using the Amphora package with default trimming. Amino acid substitution models were selected for each alignment using the ProteinModelSelection.pl script in RAxML (v. 7.3.5), which resulted in a total of six different models for the 31 alignments. Alignments were concatenated into one alignment and a best scoring ML phylogeny was computed using RAxML v. 7.3.5 with the partitioned amino acid models, estimated GAMMA model of rate heterogeneity and 1000 bootstrap replicates. This resulted in a ML phylogeny with a monophyletic outgroup and 100% bootstrap support for all nodes, highly similar to the one presented in [15].

All metagenomic sequences from the 0.1-0.8 size fraction, classified as Rickettsiales in the Amphora analysis (19,495 sequences in total; see above) were aligned together with sequences from the the eight SAR11 and seven Rickettsiales genomes to the corresponding Amphora HMM using default trimming. Metagenomic sequences consisting of all-gap columns were removed (resulting in 18,278 retained metagenomic sequences) and alignments concatenated into one global alignment with gaps inserted for metagenomic sequences where these did not match a certain alignment. Sequences from the metagenomic data were placed onto the SAR11 ML phylogeny using RAxML v. 7.3.5 with the ML method and the partitioned amino acid models estimated previously. Finally, sequences with <0.95 best-placement likelihood weights were removed, branches coloured according to sample origin and the phylogeny visualized in Archaeopteryx (v. 0.968, [16]).

### Functional analysis

Frequencies of SAR11 genes in the EMP and ED glycolytic pathways was calculated using reciprocal best blast hits as described in [17] but with the BLASTP program. SAR11 candidate genes used to search the metagenomic dataset were selected from all SAR11 genomes using the supplementary cluster annotations reported in [15]. In Ca. Pelagibacter HTCC1062, the ED query genes consisted of SAR11_0768, SAR11_0773-5 and SAR11_0777-79 while the EMP query genes consisted of SAR11_0155, SAR11_0193, SAR11_0584-86, SAR11_0927 and SAR11_0939. Amino acid sequences for 6-phosphofructokinase 1 (*pfkA*), fructose-bisphosphate aldolase (*fbaA*) and pyruvate kinase (*pyk*) from alphaproteobacterium HIMB59 and Ca. Pelagibacter HTCC9565 were also included in the EMP query.

### Analysis of SAR11-like contigs

Contigs in the combined Baltic Sea metagenomic assembly were identified as SAR11 by 1) the proportion of encoded peptides classified as SAR11 by APIS and 2) by the tetranucleotide composition of contig sequences. This included a total of 20,309 contigs, comprising 27,282,687 nucleotides, with an N50 length of 1,730 bp and a maximum length of 28,089 bp. The contigs were divided into brackish and marine bins based on the proportion of reads from brackish (GS665-GS682) or marine (GS683-GS695) samples. The frequency of individual KEGG orthologs was counted within each contig pool and normalized to the number of RecA sequences in each pool (16 marine, 18 brackish), as above.

# References

1. Zeigler Allen L, Allen EE, Badger JH, McCrow JP, Paulsen IT, et al. (2012) Influence of nutrients and currents on the genomic composition of microbes across an upwelling mosaic. ISME J 6: 1403–1414. doi:10.1038/ismej.2011.201.

2. Tanenbaum DM, Goll J, Murphy S, Kumar P, Zafar N, et al. (2010) The JCVI standard operating procedure for annotating prokaryotic metagenomic shotgun sequencing data. Stand Genomic Sci 2: 229–237. doi:10.4056/sigs.651139.

3. Rusch DB, Halpern AL, Sutton G, Heidelberg KB, Williamson S, et al. (2007) The Sorcerer II Global Ocean Sampling Expedition: Northwest Atlantic through Eastern Tropical Pacific. PLoS Biol 5: e77. doi:10.1371/journal.pbio.0050077.

4. Margulies M, Egholm M, Altman WE, Attiya S, Bader JS, et al. (2005) Genome sequencing in microfabricated high-density picolitre reactors. Nature 437: 376–380. doi:10.1038/nature03959.

5. Herlemann DP, Labrenz M, Jürgens K, Bertilsson S, Waniek JJ, et al. (2011) Transitions in bacterial communities along the 2000 km salinity gradient of the Baltic Sea. ISME J 5: 1571–1579. doi:10.1038/ismej.2011.41.

6. Seshadri R, Kravitz SA, Smarr L, Gilna P, Frazier M (2007) CAMERA: A Community Resource for Metagenomics. PLoS Biol 5: e75. doi:10.1371/journal.pbio.0050075.

7. Goll J, Rusch DB, Tanenbaum DM, Thiagarajan M, Li K, et al. (2010) METAREP: JCVI metagenomics reports--an open source tool for high-performance comparative metagenomics. Bioinforma Oxf Engl 26: 2631–2632. doi:10.1093/bioinformatics/btq455.

8. Wu M, Scott AJ (2012) Phylogenomic analysis of bacterial and archaeal sequences with AMPHORA2. Bioinforma Oxf Engl 28: 1033–1034. doi:10.1093/bioinformatics/bts079.

9. Stamatakis A (2006) RAxML-VI-HPC: maximum likelihood-based phylogenetic analyses with thousands of taxa and mixed models. Bioinformatics 22: 2688–2690. doi:10.1093/bioinformatics/btl446.

10. Berger SA, Krompass D, Stamatakis A (2011) Performance, Accuracy, and Web Server for Evolutionary Placement of Short Sequence Reads under Maximum Likelihood. Syst Biol 60: 291–302. doi:10.1093/sysbio/syr010.

11. Whelan S, Goldman N (2001) A General Empirical Model of Protein Evolution Derived from Multiple Protein Families Using a Maximum-Likelihood Approach. Mol Biol Evol 18: 691–699.

12. Sharon I, Pati A, Markowitz V, Pinter R (2009) A statistical framework for the functional analysis of metagenomes. In: Batzoglou S, editor. Research in Computational Molecular Biology. Lecture Notes in Computer Science. Vol. 5541. pp. 496–511. doi:10.1007/978-3-642-02008-7_35.

13. Giovannoni SJ, Tripp HJ, Givan S, Podar M, Vergin KL, et al. (2005) Genome Streamlining in a Cosmopolitan Oceanic Bacterium. Science 309: 1242–1245. doi:10.1126/science.1114057.

14. Oh H-M, Kang I, Lee K, Jang Y, Lim S-I, et al. (2011) Complete genome sequence of strain IMCC9063, belonging to SAR11 subgroup 3, isolated from the Arctic Ocean. J Bacteriol 193: 3379–3380. doi:10.1128/JB.05033-11.

15. Grote J, Thrash JC, Huggett MJ, Landry ZC, Carini P, et al. (2012) Streamlining and Core Genome Conservation among Highly Divergent Members of the SAR11 Clade. mBio 3.

16. Han M, Zmasek C (2009) phyloXML: XML for evolutionary biology and comparative genomics. BMC Bioinformatics 10: 356. doi:10.1186/1471-2105-10-356.

17. Schwalbach MS, Tripp HJ, Steindler L, Smith DP, Giovannoni SJ (2010) The presence of the glycolysis operon in SAR11 genomes is positively correlated with ocean productivity. Environ Microbiol 12: 490–500. doi:10.1111/j.1462-2920.2009.02092.x.
